# Supplementary material for: Patient and public involvement in numerical aspects of trials: a mixed methods theory-informed survey of trialists’ current practices, barriers and facilitators
Source: BMJ Open. 2021 Mar 18;11(3):e046977. doi: 10.1136/bmjopen-2020-046977 (PMC7978289; doi:10.1136/bmjopen-2020-046977)
Supplement: Supplementary data [file bmjopen-2020-046977supp001.pdf]

Survey
Demographic questions

Questions	Response options
**What is your age?**	18-24 years old<br>25-34 years old<br>35-44 years old<br>45-54 years old<br>55-64 years old<br>65-74 years old<br>75 years or older
**Gender: What is your gender?**	Female<br>Male<br>Other<br>Rather not say
**Geographic: Where is your workplace is based?**	England<br>North East England<br>North West England<br>Yorkshire and the Humber<br>West Midlands<br>East Midlands<br>South West England<br>South East England<br>London<br>East England<br>Northern Ireland<br>Scotland<br>Wales
**Job: What is your main role in the trial?**	Trial Manager
	Chief Investigator
	Co-Investigator
	Statistician
	Data manager

Goulao B, *et al. BMJ Open* 2021; 11:e046977. doi: 10.1136/bmjopen-2020-046977

	Patient partner
	Qualitative researcher
	Health Economist
	Don't work in a trial
	Other
**Job: How long have you worked in clinical trials?**	Less than 5 years<br>Between 5-10 years<br>More than 10 years
**Job: Which aspects of the trials process are you directly involved in?**	Trial design
	Undertaking the trial (e.g. identifying or recruiting participants, collecting data)
	Analysis and/or interpretation of results
	Dissemination of findings
	None of the above
	All of the above
	Other

Goulao B, *et al. BMJ Open* 2021; 11:e046977. doi: 10.1136/bmjopen-2020-046977

Current behavior / beliefs

Questions	Response options
How often are you involved in the numerical aspects of a trial, according to the definition above?	1: never<br>2: rarely<br>3: regularly<br>2: every day<br>0: other
Do you involve patient or public partners in the numerical aspects of trials?	1: yes<br>2: no<br>3: don't know
Can you provide the reasons for why you do, or do not, involve patient or public partners in the numerical aspects of trials?	Open question
What stage of a trial do you involve public or patient partners in the numerical aspects of a trial?	Research design
	Undertaking the research (e.g. Identifying or recruiting participants, collecting data)
	Analysis and/or interpretation of results
	Dissemination of findings
	None of the above

Goulao B, *et al. BMJ Open* 2021; 11:e046977. doi: 10.1136/bmjopen-2020-046977

	Other (please specify)
When you involve patient or public partners in the numerical aspects of a trial, where on the scale do you picture yourself?	0-100 (informing – co-producing)
When involving patient or public partners in numerical aspects, do you think the patient or public partners need to have particular experience or skills?	1: yes<br><br>2: no
If you answered "Yes" to the last questions, what kind of experience or skills are you looking for?	Open question

Theoretical domains framework questions

**Questions**	**Response options**
On a scale of 1 to 7, how familiar are you with involving patient or public partners in numerical aspects of trials ? Scale 1-7, 1 being not at all familiar and 7 being very familiar	1-7
What do you know about how to involve patient or public partners in numerical aspects of trials? Please provide examples of when this has worked well.	Open question
On a scale of 1 to 7, grade your own ability to involve patient or public partners in numerical aspects of trials? Scale 1 – 7: 1 being unable and 7 being very able	1-7
Is involving patient or public partners in numerical aspects of trials an expected role within your job? E.g. an explicit goal or objective within your job specification	1: yes<br><br>2: no<br><br>0: other

Goulao B, *et al. BMJ Open* 2021; 11:e046977. doi: 10.1136/bmjopen-2020-046977

On a scale of 1 to 7, do you feel confident in your ability to involve patient or public partners in numerical aspects of trials? Scale 1- 7, 1 being unable and 7 being very able	1-7
Do you think that involving patient or public partners in numerical aspects of trials is hard to deliver?	1: yes<br><br>2: no<br><br>0: other
If you answered "Yes", what it is that makes it hard and what could make it easier?	Open question
On a scale of 1 to 7, how much do you agree with this sentence? Involving patient or public partners in numerical aspects of trials is a good thing. Scale 1 – 7, 1 being do not agree and 7 being completely agree	
**Do you think involving patient or partners in numerical aspects of a trial can have a positive impact on the following groups?**	
• On the researcher	1: Yes – positive impact<br><br>2: No – no impact<br><br>3: No – negative impact
• On the quality of trial	1: Yes – positive impact<br><br>2: No – no impact

	3: No – negative impact
• On the patient partner	1: Yes – positive impact<br><br>2: No – no impact<br><br>3: No – negative impact
If you answer "No" to one of the questions, please specify why you think public or patient partners in numerical aspects of trials have no impact or a negative impact	Open question
What positive impacts can patient or public partners have on the numerical aspects of trials?	Open question
Do you think that there is a good balance between the challenges of involving patient or public partners in numerical aspects of trials (e.g. the difficulty of operationalization or implementation) and the potential benefits of patient or public partners involvement?	1: yes<br><br>2: no
**When you involve patient or public partners in numerical aspects of trials, do you get a reward or recognition from the following groups?**	
• Patient and public partners	1: yes<br><br>2: no
• My work peers	1: yes<br><br>2: no

• My line manager	1: yes<br><br>2: no
Please tell us who else rewards or recognizes you when you involve public and patient partners in numerical aspects of trials	Open question
Will you involve patient or public partners in numerical aspects in your next project?	1: yes definitely<br><br>2: yes probably<br><br>3: no definitely<br><br>4: no probably<br><br>5: I don't know
On a scale of 1 to 7, how much do you agree with this sentence? Working on something else on my agenda is a higher priority or more urgent than involving patient or public partners in the numerical aspects of trials.	1-7
On a scale of 1 to 7, how much do you agree with this sentence? Involving patient or public partners in numerical aspects of trials is something I do automatically.	1-7
Do you have the resources you need to involve patient or public partners in numerical aspects of trials?	1: yes<br><br>2: no
**Do you agree with these sentences?**	
• University/Employer provides training to involve patient or public partners in numerical aspects of trials.	1: yes<br><br>2: no
• Involving patient or public partners in numerical aspects of trials is compatible with daily practice	1: yes

	2: no
• Patient or public partners are motivated to get involved in numerical aspects of trials	1: yes<br><br>2: no
What in your work environment could help to involve patient or public partners in numerical aspects of trial?	Open question
What in your work environment could be a barrier to involve patient or public partners in numerical aspects of trial?	Open question
On a scale of 1 to 7, how much do you agree with this sentence? Most people who are important (E.g. co-workers, peers) think that I should involve patient or public partners in numerical aspects of trials	1-7
**From the list below, who encourages you to involve patient or public partners in numerical aspects of trials? Tick all the relevant boxes no maximum**	
• Myself	
• Co-workers	
• Manager	
• Scientific literature	
• Social media	
• My personal environment	
• Public	
• Institution	

Goulao B, *et al. BMJ Open* 2021; 11:e046977. doi: 10.1136/bmjopen-2020-046977

• Regulation	
• Mass media	
• No encouragement	
• Other	
**On the list below, who is a barrier for you involve patient or public partners in numerical aspect of trials?**	
• Myself	
• Co-workers	
• Manager	
• Scientific literature	
• Social media	
• My personal environment	
• Public	
• Institution	
• Regulation	
• Mass media	
• No encouragement	

Goulao B, *et al. BMJ Open* 2021; 11:e046977. doi: 10.1136/bmjopen-2020-046977

• Other	
Does involving patient or public partners in numerical aspects of trials lead to any of the following emotions … Tick the relevant boxes (3 boxes minimum)	
Optimism	
Ease	
Calm	
Relaxation	
Appreciation	
Happiness	
Nervousness	
Pessimism	
Depression	
Agitation	
Sadness	
Anxiety	
Do you have a clear plan on which numerical aspects of trials you should involve the patient or partners with?	1: yes<br>2: no

Do you have a clear plan on how you will involve the patient or public partners in the numerical aspects of trials?	1: yes<br><br>2: no
Do you have a clear plan on how often you will involve the patient or public partners in numerical aspects of trials?	1: yes<br><br>2: no
Is there anything that would remind you to involve patients or public partners in numerical aspects of trials?	Open question
On a scale of 1 to 7, how much do you agree with this sentence? I involve patient or public partners in numerical aspects of trials without having to consciously thinking about it. Scale 1-7, 1 being not at all and 7 being definitely	1-7
Is there anything else you would like to tell us about experiences with public involvement in numerical aspects of trial?	Open question

Goulao B, *et al. BMJ Open* 2021; 11:e046977. doi: 10.1136/bmjopen-2020-046977
