# Supplementary material for: Patient and public involvement in numerical aspects of trials: a mixed methods theory-informed survey of trialists’ current practices, barriers and facilitators
Source: BMJ Open. 2021 Mar 18;11(3):e046977. doi: 10.1136/bmjopen-2020-046977 (PMC7978289; doi:10.1136/bmjopen-2020-046977)
Supplement: Supplementary data [file bmjopen-2020-046977supp002.pdf]

Supplementary Table 1 – Demographic characteristics by respondent status (respondents were defined as participants that replied to demographic characteristics and all theoretical domains framework questions; non-respondents were those that replied to demographic questions only)

	Respondents (N=187)	Non-respondents (N=99)
**Age**		
18-24 years old	2 (1.1)	3 (3.0)
25-34 years old	62 (33.2)	27 (27.3)
35-44 years old	50 (26.7)	35 (35.4)
45-54 years old	41 (21.9)	20 (20.2)
55-64 years old	22 (11.8)	9 (9.1)
65+ years old	7 (3.7)	4 (4.0)
Rather not say	3 (1.6)	1 (1.0)
**Gender**		
Female	130 (69.5)	82 (82.8)
Male	50 (26.7)	17 (17.2)
Other	2 (1.1)	
Prefer not to say	5 (2.7)	
**Work location**		
England	17 (9.1)	10 (10.1)
North East England	7 (3.7)	3 (3.0)
North West England	14 (7.5)	7 (7.1)
Yorkshire and the Humber	13 (7.0)	11 (11.1)
West Midlands	12 (6.4)	1 (1.0)
East Midlands	7 (3.7)	6 (6.1)
South West England	11 (5.9)	6 (6.1)
South East England	19 (10.2)	10 (10.1)
London	33 (17.6)	18 (18.2)
East England	4 (2.1)	3 (3.0)
Northern Ireland	6 (3.2)	5 (5.1)
Scotland	30 (16.0)	16 (16.2)
Wales	14 (7.5)	3 (3.0)
**Which aspects of the trial process are you directly involved in?**		
Trial design	88 (47.1)	51 (51.5)
Undertaking the trial	85 (45.5)	50 (50.5)
Dissemination	89 (47.6)	39 (39.4)
Analysis	59 (31.6)	24 (24.2)
Overseeing committee	5 (2.7)	1 (1.0)
All stages	75 (40.1)	25 (25.3)
None		1 (1.0)
**Main role in trial**		
Trial manager	125 (66.8)	69 (69.7)
Chief investigator	3 (1.6)	
Statistician	29 (15.5)	8 (8.1)
Health economist	3 (1.6)	3 (3.0)
Qualitative researcher	8 (4.3)	
Patient Partner	8 (4.3)	3 (3.0)
Co-investigator	16 (8.6)	10 (10.1)

Goulao B, *et al. BMJ Open* 2021; 11:e046977. doi: 10.1136/bmjopen-2020-046977

Researcher	7 (3.7)	6 (6.1)
Systematic reviewer	1 (0.5)	
Programmer		1 (1.0)
PPI lead	3 (1.6)	4 (4.0)
Don't work in a trial		2 (2.0)
Missing	63 (33.2)	30 (30.3)
**How long have you been working in trials?**		
Less than 5 years	57 (30.5)	35 (35.4)
Between 5-10 years	61 (32.6)	28 (28.3)
More than 10 years	69 (36.9)	36 (36.4)
**When do you involve patient or public partners in numerical aspects of trials?**		
Never	7 (3.7)	7 (7.1)
Rarely	23 (12.3)	18 (18.2)
Regularly	109 (58.3)	57 (57.6)
Every day	48 (25.7)	11 (11.1)
Missing		6 (6.1)
**Do you involve patient or public partners in the numerical aspects of trials?**		
Yes	116 (62.0)	18 (18.2)
No	48 (25.7)	6 (6.1)
Don't know	23 (12.3)	3 (3.0)
Missing		72 (72.7)

Goulao B, *et al. BMJ Open* 2021; 11:e046977. doi: 10.1136/bmjopen-2020-046977

Supplementary Table 2 – Responses to the Theoretical Domains Framework questions by whether respondent said they involved patient and public partners (PPP) in numerical aspects of trials – median (percentile 25 – percentile 75), count for scale variables or n (%) out of N for binary variables

	Respondents involve PPP (N=116)	Respondents do not involve PPP or are not sure (N=71)
**TDF domain:** Knowledge		
How familiar are you with involving PPP in numerical aspects of trials?	4 (3.0-5.0),116	2 (1.0-3.0),71
**TDF domain:** Skills		
Grade your own ability to involve PPP in numerical aspects of trials	4 (3.0-5.0),116	2 (1.0-4.0),71
**TDF domain:** Social / professional role		
Is involving PPP in numerical aspects of trials an expected role within your job?	46 (39.7)	3 (4.2)
**TDF domain: Beliefs about capability**		
Do you feel confident in your ability to involve PPP in numerical aspects of trials?	4 (3.0-5.0),111	3 (2.0-4.0),62
Do you think that involving PPP in numerical aspects of trials is hard to deliver?	52 (44.8)	34 (47.9)
**TDF domain:** Optimism		
Involving PPP in numerical aspects of trials is a good thing	6 (5.0-7.0),108	5 (4.0-6.0),61
**Do you think involving patient or partners in numerical aspects of a trial can have a positive impact on the researcher?**		
Yes	96 (82.8)	43 (60.6)
No impact	5 (4.3)	11 (15.5)
Negative impact	1 (0.9)	2 (2.8)
Missing	14 (12.1)	15 (21.1)
**on the quality of the trial?**		
Yes	93 (80.2)	46 (64.8)
No impact	8 (6.9)	9 (12.7)
Negative impact	1 (0.9)	1 (1.4)
Missing	14 (12.1)	15 (21.1)
**on the patient partner?**		
Yes	95 (81.9)	50 (70.4)
No impact	6 (5.2)	4 (5.6)
Negative impact	1 (0.9)	2 (2.8)
Missing	14 (12.1)	15 (21.1)
**TDF domain:** beliefs about consequences		
There is a good balance between the challenges of involving PPP in numerical aspects of trials and the potential benefits	68 (58.6)	22 (31.0)
**TDF domain:** Reinforcement		
You get recognition from PPP, when you involve them in numerical aspects of trials	35 (30.2)	12 (16.9)
You get recognition from work peers	21 (18.1)	5 (7.0)

Goulao B, *et al. BMJ Open* 2021; 11:e046977. doi: 10.1136/bmjopen-2020-046977

You get recognition from your manager	23 (19.8)	6 (8.5)
**TDF domain:** Intention		
**Will you involve PPP in numerical aspects of your next project?**		
Yes definitely	41 (35.3)	4 (5.6)
Yes probably	42 (36.2)	16 (22.5)
No definitely	0	1 (1.4)
No probably	4 (3.4)	9 (12.7)
I don't know	12 (10.3)	24 (33.8)
Missing	17 (14.7)	17 (23.9)
**TDF domain:** Goals		
Working on something else on my agenda is a higher priority than involving PPP in the numerical aspects of trials	4 (3.0-5.0),99	5 (4.0-6.0),54
**TDF domain:** Memory, attention, decision process		
Involving PPP in numerical aspects of trials is something I do automatically	4 (3.0-6.0),93	2 (1.0-2.5),52
**TDF domain:** Environmental context and resources		
Have the resources needed to involve PPP in numerical aspects of trials	55 (47.4)	16 (22.5)
Employer provides support to involve PPP in numerical aspects of trials	47 (40.5)	13 (18.3)
Employer provides training to involve PPP in numerical aspects of trials	31 (26.7)	7 (9.9)
Involving PPP in numerical aspects of trials is compatible with daily practice	63 (54.3)	17 (23.9)
PPP are motivated to get involved in numerical aspects of trials	59 (50.9)	14 (19.7)
**TDF domain:** Social influences		
Most people who are important think I should involve PPP in numerical aspects of trials	5 (4.0-6.0),94	3 (1.0-4.0),50
**Who encourages you to involve PPP in numerical aspects?**		
Myself	63 (54.3)	15 (21.1)
Co-workers	45 (38.8)	11 (15.5)
Manager	44 (37.9)	10 (14.1)
Social media	14 (12.1)	6 (8.5)
My personal environment	18 (15.5)	1 (1.4)
Scientific literature	31 (26.7)	15 (21.1)
Public	24 (20.7)	9 (12.7)
Institution	38 (32.8)	10 (14.1)
Regulation	22 (19.0)	15 (21.1)
Mass media	3 (2.6)	3 (4.2)
No encouragement	7 (6.0)	14 (19.7)
**Who is a barrier for you to involve PPP in numerical aspects?**		
Myself	6 (5.2)	7 (9.9)
Co-workers	4 (3.4)	7 (9.9)

Goulao B, *et al. BMJ Open* 2021; 11:e046977. doi: 10.1136/bmjopen-2020-046977

Manager	6 (5.2)	10 (14.1)
Scientific literature	2 (1.7)	3 (4.2)
Social media	2 (1.7)	
My personal environment	11 (9.5)	6 (8.5)
Public	5 (4.3)	7 (9.9)
Institution	13 (11.2)	13 (18.3)
Regulation	10 (8.6)	5 (7.0)
Mass media	5 (4.3)	3 (4.2)
No barrier	47 (40.5)	13 (18.3)
**TDF domain:** Emotions		
**Emotions related to involving PPP in numerical aspects**		
Optimism	65 (56.0)	29 (40.8)
Ease	18 (15.5)	5 (7.0)
Calm	14 (12.1)	3 (4.2)
Relaxation	3 (2.6)	3 (4.2)
Appreciation	71 (61.2)	32 (45.1)
Nervousness	37 (31.9)	18 (25.4)
Pessimism	7 (6.0)	12 (16.9)
Depression		2 (2.8)
Agitation	9 (7.8)	11 (15.5)
Happiness	27 (23.3)	8 (11.3)
Sadness	2 (1.7)	2 (2.8)
Anxiety	25 (21.6)	17 (23.9)
**TDF domain:** Behavioural regulation		
**Do you have a clear plan on…**		
Which numerical aspects of trials you should involve PPP with?	33 (28.4)	5 (7.0)
How you will involve PPP in numerical aspects of trials?	36 (31.0)	6 (8.5)
How often will you involve PPP in numerical aspects of trials?	37 (31.9)	4 (5.6)
**TDF domain:** Nature of behaviour		
Involve PPP in numerical aspects of trials without consciously thinking about it	4 (2.0-5.0),90	2 (1.0-3.5),48

Goulao B, *et al. BMJ Open* 2021; 11:e046977. doi: 10.1136/bmjopen-2020-046977
