# Supplementary material for: Patient and public involvement in numerical aspects of trials: a mixed methods theory-informed survey of trialists’ current practices, barriers and facilitators
Source: BMJ Open. 2021 Mar 18;11(3):e046977. doi: 10.1136/bmjopen-2020-046977 (PMC7978289; doi:10.1136/bmjopen-2020-046977)
Supplement: Author's manuscript [file bmjopen-2020-046977.draft_revisions.pdf]

BMJ Open

BMJ Open is committed to open peer review. As part of this commitment we make the peer review history of every article we publish publicly available.

When an article is published we post the peer reviewers' comments and the authors' responses online. We also post the versions of the paper that were used during peer review. These are the versions that the peer review comments apply to.

The versions of the paper that follow are the versions that were submitted during the peer review process. They are not the versions of record or the final published versions. They should not be cited or distributed as the published version of this manuscript.

BMJ Open is an open access journal and the full, final, typeset and author-corrected version of record of the manuscript is available on our site with no access controls, subscription charges or pay-per-view fees (http://bmjopen.bmj.com).

If you have any questions on BMJ Open's open peer review process please email info.bmjopen@bmj.com

BMJ Open

Patient and public involvement in numerical aspects of trials: a theory-informed survey of trialists current practices, barriers and facilitators

Journal:	*BMJ Open*
Manuscript ID	bmjopen-2020-046977
Article Type:	Original research
Date Submitted by the Author:	14-Nov-2020

Complete List of Authors:	Goulao, Beatriz; University of Aberdeen, Health Services Research Unit<br>Poisson, Camille; University of Aberdeen, Health Services Research Unit<br>Gillies, Katie; University of Aberdeen, Health Services Research Unit

Keywords:	Clinical trials < THERAPEUTICS, STATISTICS & RESEARCH METHODS, HEALTH SERVICES ADMINISTRATION & MANAGEMENT

SCHOLARONE™
Manuscripts

I, the Submitting Author has the right to grant and does grant on behalf of all authors of the Work (as defined in the below author licence), an exclusive licence and/or a non-exclusive licence for contributions from authors who are: i) UK Crown employees; ii) where BMJ has agreed a CC-BY licence shall apply, and/or iii) in accordance with the terms applicable for US Federal Government officers or employees acting as part of their official duties; on a worldwide, perpetual, irrevocable, royalty-free basis to BMJ Publishing Group Ltd ("BMJ") its licensees and where the relevant Journal is co-owned by BMJ to the co-owners of the Journal, to publish the Work in this journal and any other BMJ products and to exploit all rights, as set out in our licence.

The Submitting Author accepts and understands that any supply made under these terms is made by BMJ to the Submitting Author unless you are acting as an employee on behalf of your employer or a postgraduate student of an affiliated institution which is paying any applicable article publishing charge ("APC") for Open Access articles. Where the Submitting Author wishes to make the Work available on an Open Access basis (and intends to pay the relevant APC), the terms of reuse of such Open Access shall be governed by a Creative Commons licence – details of these licences and which Creative Commons licence will apply to this Work are set out in our licence referred to above.

Other than as permitted in any relevant BMJ Author's Self Archiving Policies, I confirm this Work has not been accepted for publication elsewhere, is not being considered for publication elsewhere and does not duplicate material already published. I confirm all authors consent to publication of this Work and authorise the granting of this licence.

For peer review only

Patient and public involvement in numerical aspects of trials: a theory-informed survey of trialists current practices, barriers and facilitators

Beatriz Goulao[1], beatriz.goulao@abdn.ac.uk, corresponding author

Camille Poisson[2], hsru.aberdeen@abdn.ac.uk

Katie Gillies[1], k.gilles@abdn.ac.uk

1 – Health Services Research Unit, University of Aberdeen

Keywords: Patient and public involvement; clinical trials; trial methodology; theoretical domains framework

Abstract (261/300)

Background: Patient and Public Involvement (PPI) is increasingly common in UK trials and acknowledged as a way to increase research relevance as well as a requirement by funders. Numerical aspects are crucial in the design, conduct, analysis and dissemination of trials, however the extent to which patient and public partners have a say is unknown.

Objective: We aimed to find out if trialists involve patients and the public in numerical aspects of trials, how and what are the barriers and facilitators to doing it.

Design: We developed a survey based on the theoretical domains framework. We used a mixed methods approach to analyse the data and to identify important domains.

Setting and participants: Our survey included UK based trialists.

Main outcome measures: Trialists' behaviour of involving patients and the public in numerical aspects of trials and its determinants

Results: We included 187 responders. The majority were female (70%), trial managers (67%) and involved public and patient partners in numerical aspects of trials (60%). We found lack of knowledge, trialists' perception of public and patient partners' skills, capabilities and motivations, scarce resources, lack of reinforcement, and lack of guidance were barriers to involving public and patient partners in numerical aspects of trials. Positive beliefs about consequences were an incentive to doing it.

Conclusions: More training, guidance and funding can help trialists involve patient and public partners in numerical aspects, although they were uncertain about public and patient partners' motivation to be involved. Future research should focus on finding public and patient partners' motivations, and develop strategies to improve communication of numerical aspects.

Summary of the article

Strengths and limitations of this study

- First investigation of trialists practice regarding involving patients and the public in numerical aspects of trials and aimed at UK based trialists
- We achieved a wide representation of trialists working in clinical trials in the UK

For peer review only - http://bmjopen.bmj.com/site/about/guidelines.xhtml

- We describe current practice as well as determinants of behaviour using a theory-informed approach based on the theoretical domains framework
- Respondents' interpretation of involving patients and the public numerical aspects varied, so the results apply to a spectrum of behaviours. Respondents are likely to have stronger views of patient and public involvement (PPI) and PPI in numerical aspects of trial since they opted to answer our survey.

For peer review only

For peer review only - http://bmjopen.bmj.com/site/about/guidelines.xhtml

Background

Patient and Public Involvement (PPI) has been defined as “research being carried out ‘with’ or ‘by’ members of the public (including patients and carers) rather than ‘to’, ‘about’ or ‘for’ them.” (http://www.invo.org.uk/) PPI activity has become increasingly common in trials in the United Kingdom (UK) for several reasons: its supporters believe it makes research more relevant and it is, morally, the right thing to do. It is also a requirement from major funders, including the National Institute for Health Research (NIHR).[1]

In a trial context, patient and public partners have been primarily involved in agenda setting, steering committees, ethical review, protocol development, and piloting [2] but PPI remains seen as tokenistic and a ticking box exercise in many settings [3], [4]. Trials are primarily quantitative research and often key decisions are based on numerical aspects and their discussion. However, the extent to which patients and the public get involved in those discussions is unknown with some evidence suggesting communication of statistical aspects of trials needs to be clearer in order to achieve meaningful involvement [5]. At the same time, involving patients and the public in statistical and health economics aspects of trials can result in enriching data interpretation, better research, with more robust and implementable evidence [6], [7].

Patient and public involvement can be considered a professional behavioural since it involves a series of actions to accomplish a goal (such as inviting a patient partner to contribute to a discussion). To understand a specific behaviour and its determinants it can be helpful to use behavioural theoryto provide a systematic and replicable framework. The Theoretical Domains Framework (TDF) was developed to focus on healthcare professional behaviour, specifically around evidence-based medicine and the adoption of new practices and is made of up of 15 behavioural domains [8]. Previous work has used the TDF to explore researchers’ beliefs related to PPI in health research (Boylan), but to our knowledge this is the first application of the framework to explore PPI in trials and, in particular, PPI in numerical aspects of trials. We aimed to use a theory-informed approach to find out whether trialists involve patient and public partners in numerical aspects of trials and what are the barriers and facilitators to doing it.

Methods

We conducted an online survey approved the College of Ethics Research Committee (CERB/2019/4/1759) of the University of Aberdeen.

Survey design

The survey was divided into the following sections:

- Demographic questions
- Definition of numerical aspects of trials
- General questions about PPI involvement in numerical aspects of trials
- Theoretical domain framework questions

Demographic questions focused on characterising our sample (age, gender, trial work experience and role). We agreed the definition of “Numerical aspects of trials” ("Any aspects of a trial where people measure (or plan to measure), manage, analyse or share information that is presented as one or more numbers.") through an iterative process involving thirteen members of the Health Services Research

For peer review only - http://bmjopen.bmj.com/site/about/guidelines.xhtml

Unit (HSRU) at the University of Aberdeen (including a variety of different roles in the unit, such as a Professor of Health Services Research, a health economist, a statistician, trial manager, PPI/engagement co-ordinator, research fellow, assistant researcher, and quality assurance manager). The definition was included in the survey. Responders were asked about their current practice regarding involving public and patient partners in numerical aspects of trials.

The TDF was developed as a theoretical framework and includes behavioural theories and constructs and proposes that determinants of professionals' behaviour can be clustered in "domains". The TDF, after update from Presseau et al [9], consists of fifteen domains. The TDF section of our survey was developed based on previous surveys [10], [11] and was reviewed by the project team and by an expert in TDF research (ED).

The survey was piloted by eight staff or student members from the University of Aberdeen. The survey was administered and delivered through Survey Monkey. Informed consent was requested and embedded in the survey. The final version contained 43 questions consisting of both closed and open questions (see **Appendix 1 – questionnaire**). The participants were able to withdraw from the survey at any time.

Target population

The target population was any stakeholder working in clinical trials (from here onwards called a "trialist"), for example: trial managers, principal or chief investigators, statisticians, clinicians, patient or public partners, PPI coordinators, research fellows. To take part in our study, the participants needed to be 18 years old or over, to work in a UK clinical trial's unit, to understand English and to agree to take part in the study.

Participant recruitment

The questionnaire was disseminated using an online link via email and social media. We used a snowball approach to recruit survey responders by disseminating the survey to colleagues, known networks, and through social media (LinkedIn, Twitter, Facebook and Instagram). We used common hashtags (#) and pictures to encourage responses. We contacted stakeholders via professional mailing lists to ask to disseminate our survey, such as: PPI coordinator's mailing lists, clinical trial units (CTU) websites and UK Clinical Research Collaboration. The survey link was active from the 11th of June 2019 to the 1st of July 2019.

Survey analysis

The survey analysis was divided in two parts: analysis of the closed-ended questions (as quantitative data) and the analysis of open questions (as qualitative data). We exported the data into Stata and generated statistical summaries of the closed-ended questions. The Likert scale questions used a scale from 1 (weaker agreement / confidence / etc) to 7 (stronger agreement / confidence / etc). They were presented using medians, interquartile ranges and counts. Binary questions (with only two possible answers, for example yes/no) are presented as n (%) out of the available answers. We split results into respondents that self-report to perform the behaviour assessed (involving public and patient partners in numerical aspects of trials) and those that did not or were not sure. When questions included a category named "other", the answers given were grouped together, coded and counted. Analysis of open-ended questions was done using a deductive thematic analysis approach informed by the TDF by one author (BG). If data did not fit into the pre-specified TDF domains, an inductive approach was

For peer review only - http://bmjopen.bmj.com/site/about/guidelines.xhtml

taken. We compared the quantitative and qualitative data to identify the most salient domains of the TDF that explained the behaviour. This process was done by one author (BG) and reviewed using a subset of qualitative data by a second author (KG). We did not undertake any statistical testing since our aim was to describe the data and not to test hypotheses. We used Stata 15 for the purposes of summarising quantitative data and Microsoft Word to analyse qualitative data.

Identifying the most "relevant" domains to involve patients and the public in numerical aspects of trials

In order to establish the barriers and facilitators of the target behaviour (patient and public involvement in numerical aspects of trials), criteria were developed to determine which domains of the TDF were 'relevant' for this behaviour based on previous TDF research [12]. Relevant domains were those that were considered to show variation in reported behaviour/beliefs between trialists that involve patients and the public in numerical aspects and those that don't.

Results

A total of 317 people accessed the survey. From those 317, 31 did not provide any information and were not included in the analysis. A further 99 participants did not respond to the TDF focussed questions. Therefore, the 187 participants that replied to at least one TDF question were included in the final analysis (**Figure 1**). **Supplementary table 1** provides demographic information based on respondent status. Respondents and non-respondents had similar features with a few exceptions: there was a higher percentage of women in the group of non-respondents compared with respondents (n=82, 83 vs n=130, 70%); there was a higher percentage of respondents involved in all stages of trial (n=75, 40%) compared with non-respondents (n=25, 25%), however the most common trial stages that respondents and non-respondents were involved in were the same: trial design (around 50%) and undertaking the trial (around 50%). There was a higher proportion of statisticians in the respondents' group (n=29, 16%) compared with non-respondents (n=8, 8%). A larger percentage of respondents reported to involve patient and public partners in numerical aspects of trials daily compared with non-respondents (n=48, 26 vs n=11, 11%).

Characteristics of respondents

Table 1 shows characteristics of respondents. Seventy per cent were women (n=130); respondents were based across the UK with the highest percentage coming from London (n=33, 18%). Around 40% were involved in all stages of a trial (n=75) and about half were involved in design; conduct; dissemination. One third were involved in the analysis of a trial. Most respondents were trial managers (n=125, 67%); eight respondents (4%) were patient partners in trials. About 37% (n=69) had been working in trials for more than 10 years and 31% for less than 5 (n=57).

Respondents current practice of involving public and patient partners in numerical aspects of trials

Around 62% of respondents said they involve public and patient partners in numerical aspects of trials (n=116); 26% said they did not (n=48) and 12% said they were not sure (n=23). From the trialists involving public and patient partners in numerical aspects of trials, 24% were working in the field for less than 5 years (n=28) versus 41% that did not involve public and patient partners in numerical aspects of trials (n=29). Some respondents were uncertain about what involving public and patient partners in numerical aspects meant, with definitions ranging from any discussion about numbers to involvement in the statistical analysis.

For peer review only - http://bmjopen.bmj.com/site/about/guidelines.xhtml

The most common stages of trials to involve public and patient partners in numerical aspects were trial design (n=86, 74%) and dissemination (n=84, 72%). About half involved public and patient partners in the analysis and interpretation. Respondents exemplified how they involved public and patient partners in numerical aspects: in management and steering committees where numerical data is discussed; sample size calculations and, specifically, discussing effect sizes, clinically important differences and non-inferiority margins; dissemination of results and aiding to increase clarity of communication with trial participants; grant applications and trial design stage; when and how to collect data; defining outcomes. Comments made about involving public and patient partners in the statistical analysis came from those that didn't see an interest from public and patient partners in getting involved.

Domains influencing trialists in involving patients and the public in numerical aspects of trials

TDF domains selected as the most relevant for the target behaviour are presented in **Table 2**; all TDF domains results are presented in **Supplementary Table 2**. **Table 3** summarises themes identified from the responses to open questions. By comparing the quantitative and qualitative data, we identified knowledge, skills, beliefs about capabilities, beliefs about consequences, reinforcement, environmental context and resources, social influences and behavioural regulation as being salient TDF domains. We identified an additional theme within the data on "trial communication culture" that did not fit within the TDF domains. Each of the salient TDF domains and the additional theme are presented below.

Knowledge (awareness of involving patients and the public in numerical aspects of trials)

Respondents who involved public and patient partners in numerical aspects scored higher in the knowledge domain (median 4 vs 2 out of 7 (very familiar with PPI in numerical aspects of trials) for those that did and did not involve public and patient partners in numerical aspects, respectively). Knowledge was highlighted as a barrier to involving public and patient partners in numerical aspects of trials in the open questions where respondents discussed their lack of understanding of how or when to involve public and patient partners and suggesting training as a solution. Responders highlighted public and patient partners' lack of understanding of numerical aspects or trial processes as a barrier and suggested solutions to overcoming it such as the use of visual aids or clear language.

"[What would remind me of doing PPI in numerical aspects of trials is…] A better understanding of what they can bring to other numerical aspects (other that oversight committees)." [Trial Manager ™, involves]

"Visual aids (e.g. graphs) are useful. Someone on hand to answer questions and to help interpret the information provided." [Trial Manager, involves]

Skills (ability or proficiency to involve patients and the public in numerical aspects of trials)

Respondents who involved public and patient partners in numerical aspects scored higher in the skills domain (4 vs 3 out 7 (feeling able to involve public and patient partners in numerical aspects) for those that involve and those that don't, respectively). However, the answers to open questions relevant for skills focused on patients and the publics lack of appropriate skills as a cause of concern and barrier to involve them in numerical aspects of trials.

For peer review only - http://bmjopen.bmj.com/site/about/guidelines.xhtml

"[What makes involving public and patient partners in numerical aspects hard is public and patient partners…] Lack of numeracy, analytical skills & current affairs knowledge" [*Job role not reported, involves*]

Half of the respondents believed public and patient partners need to have a set of experiences or skills to be involved in numerical aspects of trials (44% (n=52)) of trialists that involve public and patient partners in numerical aspects believed this versus 58% of those that do not (n=41)). When asked to specify what skills public and patient partners would need, some respondents focused on general skills like lived experience of disease or communication. Other respondents thought public and patient partners had to have technical skills in order to be involved, such as numeracy or mathematical skills, statistical understanding, data interpretation and understanding of graphs, knowledge of trial methodology, trial processes and research in general.

Social or professional role (perception of own role and responsibilities in relation to involving patients and the public in numerical aspects of trials)

Forty percent of respondents that involved public and patient partners in numerical aspects considered it an expected role within their jobs (n=46) vs 4% of those that did not involve public and patient partners (n=3). This was supported by qualitative data that showed that some trialists felt like other colleagues, including the Chief Investigator or statistician, would have to drive this process.

"[A barrier to involving public and patient partners in numerical aspects of trials is…] It is not spoken about much and does not always relate directly to job roles." [Statistician, not sure about involvement]

Beliefs about capability (self-belief or confidence in the ability to involve patients and the public in numerical aspects of trials)

Beliefs about trialists' own capability did not seem to drive behaviour with, for example, a similar percentage of responders finding the task difficult in those that did (n=52, 45%) and those that did not (n=34, 48%) involve public and patient partners. However, responses to open questions highlighted trialists' beliefs about public and patient partners capability as a barrier suggesting public and patient partners feel intimidated by numbers, do not grasp them or do not feel confident enough to comment.

"[Two public and patient partners are involved in sample size considerations and recruitment figures…] Both are already confident with numbers/figures so only needed some support with specifics of presentation/meaning." [Trial Manager , involves]

Beliefs about consequences (beliefs about outcomes of involving patients and the public in numerical aspects of trials)

More than half of respondents that involved public and patient partners in numerical aspects believe there is a good balance between challenges and benefits of doing so (n=68), whereas only 30% shared that belief in those that did not involve (n=22). Responses from open questions showed suggestions of positive consequences: focused on the project (such as improving the interpretation of results, data collection tools and processes, dissemination, trial recruitment, understanding of missing data, interpretation of clinical significance) or the public and patient partners (leads to better understanding of the data and trial processes, more motivated public and patient partners and better team communication). Some responders suggested negative or no consequences such as the impact on

For peer review only - http://bmjopen.bmj.com/site/about/guidelines.xhtml

public and patient partners being unclear or stressful; a disbelief that it can result in improved trial quality; and extra work and responsibilities for the researcher.

"[Involving public and patient partners in numerical aspects of trials can have…] Multiple impacts, most importantly allowing trial teams to best understand how the key messages of a study can be translated directly to those patients living with condition under study." [Statistician, involves]

Reinforcement (whether there is external stimulus to involve patients and the public in numerical aspects of trials)

A third of responders believed there was recognition from public and patient partners when involving them in numerical aspects of trials if they did so already (n=35) vs 17% for respondents that did not (n=12). Data from open questions supported these observations with trialists mentioning a lack of tangible rewards or encouragement as barriers to doing involving public and patient partners in numerical aspects.

Environmental context and resources (factors related to the work setting that influence involving patients and the public in numerical aspects of trials)

Less than half of trialists felt like they had the resources to support public and patient involvement in numerical aspects if they were doing it (n=55, 47%) and only 23% (n=16) if they were not. This was illustrated in several replies to the open questions with respondents suggesting more resources (financial, human) could facilitate involvement, as well as support identifying interested public and patient partners.

Social influences (perception of how others see one's role and how it impacts upon the ability to involve patients and the public in numerical aspects of research)

Ninety four per cent of those that involve public and patient partners in numerical aspects believe there is some type of encouragement from others to do so (n=7) versus 80% of those that do not involve public and patient partners (n=14). Forty percent of those that involve public and patient partners believe that no one acted as a barrier to do the target behaviour (n=47) vs 18% of those that do not involve public and patient partners (n=13). Qualitative data showed support from colleagues as a team effort, encouragement from management or from public and patient partners were important factors in involving them in numerical aspects of trials.

"Operationally, I don't think PPI members really add a great deal. I also don't think they tend to be interested in those [numerical] aspects. This may be because of the public perception of data and statistics as being hard/obtuse/esoteric." [TM & statistician, involves]

Behavioural regulation (perception of guidance or tools to manage or change patient and public involvement in numerical aspects of trials)

About a third of those that involve public and patient partners in numerical aspects state they have a clear plan about which aspects to select, how and when compared with around 7% of those that do not involve public and patient partners. Replies to open questions showed trialists suggest the need for clear guidance on how to conduct PPI in numerical aspects could help facilitate the behaviour, as well as having it as a requirement on ethics committees or standard operating procedures.

For peer review only - http://bmjopen.bmj.com/site/about/guidelines.xhtml

"… REC [Research ethics committee] could remind when providing the final report to consider this. The Funder often requires a report so they too could advise to do this. Being reminded by bodies that you have a regulatory or contractual obligation would help. May be journals could advise that this is done in their Instructions to Authors. The RDS also has PPI representatives on their teams and they could act in a capacity to remind people to do this. Internally in my unit, we could do with a guidance document about this." [Trial Manager, involves]

Trial communication culture

Several comments from trialists talked to a broader issue with trial communication culture that highlighted the challenges with communicating clearly about numerical aspects with the entire trial team and ensuring everyone, including patient partners, fully understand them.

"Numbers are presented to the PPI partners all the time in every trial ... So they are involved, but it is by no means actually clear if they or indeed anybody else on the trial team actually understands the figures and the way they are presented. Are they actually any different from anybody else on the trial team? Would improve transparency of decision making which I think would be an improvement even if the actual decisions within the study do not qualitatively change" [Chief Investigator, involves]

Discussion

We have conducted a large UK-based survey to identify current practice, barriers and facilitators of involving public and patient partners in numerical aspects of trials using a behavioural perspective. Because numerical aspects are central to the design, conduct, analysis and dissemination of trials our study explicitly asks about involvement in such aspects rather than more broadly. Our sample represented a wide range of ages, professions and geographic regions in the UK. We used a theory-informed approach to design and analyse our findings to identify barriers and facilitators to involvement of public and patient partners in numerical aspects of trials. We found, in general, trialists believe involving public and patient partners in numerical aspects of trials is a good thing to do but reported several barriers to doing it.

Around 60% of respondents to the survey reported to involve public and patient partners in numerical aspects of trials. This contrasts with a higher proportion of trialists involving public and patient partners in surgical trials (92%,[4]) or even in all trials, ten or more years ago (75%, [5]). Most of our respondents involved public and patient partners in numerical aspects at the trial design (68%) or dissemination (63%) stages with a significant percentage of respondents involving public and patient partners in undertaking the trial (44%) and in its analysis (35%). Crocker et al survey of PPI in surgical trials [4] and Raza et al. audit of trials in the Integrated Research Application System (IRAS) [13] showed very similar patterns, although both observed a lower prevalence of involvement in the undertaking of a trial. Since involving patients and public partners in oversight committees is common practice [2] but was not a stand-alone option in our survey, it is possible respondents included it as part of trial conduct.

There is scope to improve trialists' knowledge, environmental context and resources and behavioural regulation regarding involving public and patient partners in numerical aspects of trials. Common reflections from the open questions revealed the need for training, more funding, and resources such as finding public and patient partners that are interested in being involved in numerical aspects of trials; developing guidelines about how and when public and patient partners can be involved in

For peer review only - http://bmjopen.bmj.com/site/about/guidelines.xhtml

numerical aspects would be a natural next step. Trialists have also reported low recognition from public and patient partners, work peers and their managers. These findings are in line with other studies focused on general PPI in research [3], [14] and with wider PPI training needs requested by researchers working in clinical trials in the UK [15]. However, this need was bigger in trialists that did not involve public and patient partners in numerical aspects of trials.

Responders to the survey named more accessible and implementable findings, the opportunity of questioning researchers and more transparent partnerships between researchers and public and patient partners as benefits of involving public and patient partners in numerical aspects of trials. However, some responders saw this as an extra time-consuming task with no evidence of improving the decision making in trials and/or having an impact of patient partners. This is reflected in numerous other studies about researchers' motivations to do PPI and the ongoing debate about its impact [3], [16].

It is unclear who should be responsible for the involvement in numerical aspects of trials, given chief investigators, statisticians and health economists traditionally have the role of analysing or sharing trial data and results but trial managers are more often in charge of delivering PPI. This perception was echoed in the qualitative data where junior researchers, trial managers and qualitative researchers suggested it was not their responsibility or it was difficult for them to involve public and patient partners in numerical aspects of trials. This was a clearly important factor for responders to perform the behaviour, since only three respondents (4%) considered involving public and patient partners in numerical aspects of trials as part of their role from trialists that were not currently performing the behaviour (vs 40% of those that did it).

There is uncertainty about public and patient partners' motivation to get involved in numerical aspects of research. Around half of the respondents that involved public and patient partners in numerical aspects and 20% of trialists that don't perceived public and patient partners has being motivated to get involved. The thematic analysis identified trialists perceived patient partners lack of interest or finding numbers "boring" as a barrier to involving them in numerical aspects of trials. Our survey included only four public and patient partners respondents and, therefore, it cannot draw conclusions from their perspective but there is scope for more research understanding public and patient partners' expectations when it comes to involvement in numerical aspects of trials. However, it seems unlikely trialists could confidently speak on behalf of their public and patient partners in terms of motivation to get involved in specific trial aspects. This is because a key challenge raised by several open question comments is the lack of definition of expectations about public and patient partners from the start. Defining expectations is not common practice, even though it is considered useful by all stakeholders [4], [15].

Tokenism and the idea that public and patient partners can only contribute to a limited number of tasks related to increasing readability may be a significant barrier to involving public and patient partners in numerical aspects of trials. A lot of respondents perceived numeracy and statistical knowledge as essential for public and patient partners to get involved in numerical aspects of RCTs and, in some cases, advanced knowledge about sample size calculations and analysis. This contrasts with the fact that public and patient partners often get involved in qualitative research and reinforces perceptions of disciplinary hierarchies [3], but could also be related to the fact that PPI and qualitative research have many common features and therefore seems a natural crossover [17]. In fact, Boylan et al. reported an assumption from UK researchers that the involvement of patients in qualitative

For peer review only - http://bmjopen.bmj.com/site/about/guidelines.xhtml

interviewing and analysis is uncontroversial; some of our respondents have a clearly different view when it comes to public and patient partners involvement in quantitative analysis. From the public and patient partners perspective, a respondent pointed out that they often felt underestimated and another that tokenism is the main barrier to involving public and patient partners in numerical aspects. Wider literature on the topic shows there are appropriate methods to involve non-experts in discussions about statistical models and numbers [6], although this is a controversial topic [7]. Either way, communication, in particular about numerical aspects of trials, needs to improve to enable meaningful PPI in trials and this has been known for almost a decade [5] and it is a key priority for future PPI research according to multiple stakeholders [18].

Like with most behaviours, involving public and patient partners in numerical aspects of trials is a behaviour in a spectrum. Respondents to our survey interpreted it in a variety of ways – ranging from presenting numbers in reports to public and patient partners at trial steering meetings to involving them in doing the statistical analysis. Despite the iterative development of a definition endorsed by multiple trial stakeholders and its presentation at the beginning of the survey, it is likely that the barriers and facilitators presented here regard a range of behaviours in that spectrum. Qualitative analysis was based on open questions in a survey which are limited in their scope when compared to conducting interviews and may lead to misinterpretation of answers [19]. We tried to minimise this by double assessing a subsample of qualitative data. Our sample was diverse and coming from multiple locations and professions across the country. Respondents that answered demographic questions but did not proceed to answer questions specifically about PPI in numerical aspects of trials were mostly similar to those that did; however, we are likely to have respondents that have stronger views of PPI and PPI in numerical aspects of trial since they opted to answer our survey.

We found a generally positive attitude towards involving public and patient partners in numerical aspects of trials with 60% of the respondents reporting attempting to do so in a variety of ways. However, there is a need to understand public and patient partners expectations when getting involved in a trial and whether they would have an interest in being involved in numerical aspects. To achieve that, public and patient partners first need to be aware of the different stages of trials and the opportunities to get involved. Numerical aspects are a key element in trials and, therefore, should at least be part of an initial discussion. Our survey has also highlighted that communication of numerical and methodological aspects of trials can be challenging, not just for public and patient partners but for the whole trial team. Improving it should be a matter of providing training on either communication skills, technical aspects or both and it should be available for the whole team, including but not limited to public and patient partners. A training set co-designed by relevant stakeholders and available to all clinical trials units in the UK, including successful case studies of involving public and patient partners in numerical aspects of trials, is an essential step forward, as well as guidance on what are the most relevant numerical aspects of trials to involve public and patient partners in and how to do that in a meaningful way.

Acknowledgments: We would like to thank staff and student members at the University of Aberdeen who helped pilot test the survey and, in particular, to Dr Eilidh Duncan for her expertise and support in the development of the questions underpinned by the theoretical domains framework. We would like to acknowledge all trialists that took the time to reply to this survey and everyone that helped disseminate it.

For peer review only - http://bmjopen.bmj.com/site/about/guidelines.xhtml

Author Contributors: CP wrote the first draft of the protocol. BG and KG contributed to the design of the project and development of the protocol. BG and CP conducted the quantitative analysis; BG conducted the qualitative analysis; KG checked a sample of BG's analysis as a quality check. BG wrote the first of the manuscript. All authors approved the final version of the manuscript.

Funding: The first author was supported to develop this research by the Wellcome Trust – Institutional Strategic Support Fund at the University of Aberdeen.

Conflicts of interest: The authors have no conflicts of interest to declare.

Patient consent form: Not applicable

Data availability statement: Data is available from the corresponding author upon reasonable request.

Word count: 4940

Exclusive licence

I, the Submitting Author has the right to grant and does grant on behalf of all authors of the Work (as defined in the below author licence), an exclusive licence and/or a non-exclusive licence for contributions from authors who are: i) UK Crown employees; ii) where BMJ has agreed a CC-BY licence shall apply, and/or iii) in accordance with the terms applicable for US Federal Government officers or employees acting as part of their official duties; on a worldwide, perpetual, irrevocable, royalty-free basis to BMJ Publishing Group Ltd ("BMJ") its licensees and where the relevant Journal is co-owned by BMJ to the co-owners of the Journal, to publish the Work in BMJ Open and any other BMJ products and to exploit all rights, as set out in our licence.

The Submitting Author accepts and understands that any supply made under these terms is made by BMJ to the Submitting Author unless you are acting as an employee on behalf of your employer or a postgraduate student of an affiliated institution which is paying any applicable article publishing charge ("APC") for Open Access articles. Where the Submitting Author wishes to make the Work available on an Open Access basis (and intends to pay the relevant APC), the terms of reuse of such Open Access shall be governed by a Creative Commons licence – details of these licences and which Creative Commons licence will apply to this Work are set out in our licence referred to above.

References

[1] National Institute for Health Research, "Patient and Public Involvement in Health and Social Care Research: A handbook for researchers," pp. 1–40, 2013.

[2] A. Price *et al.*, "Patient and public involvement in the design of clinical trials: An overview of systematic reviews," *J. Eval. Clin. Pract.*, vol. 24, no. 1, pp. 240–253, 2018, doi: 10.1111/jep.12805.

[3] A. M. Boylan, L. Locock, R. Thomson, and S. Staniszewska, "'About sixty per cent I want to do it': Health researchers' attitudes to, and experiences of, patient and public involvement (PPI)—A qualitative interview study," *Heal. Expect.*, vol. 22, no. 4, pp. 721–730, 2019, doi: 10.1111/hex.12883.

For peer review only

[4] J. C. Crocker *et al.*, "Patient and public involvement (PPI) in UK surgical trials: A survey and focus groups with stakeholders to identify practices, views, and experiences," *Trials*, vol. 20, no. 1, pp. 1–15, 2019, doi: 10.1186/s13063-019-3183-0.

[5] C. Gamble *et al.*, "An evidence base to optimise methods for involving patient and public contributors in clinical trials: a mixed-methods study," *Heal. Serv. Deliv. Res.*, vol. 3, no. 39, pp. 1–142, 2015, doi: 10.3310/hsdr03390.

[6] A. Hannigan, "Public and patient involvement in quantitative health research: A statistical perspective," *Heal. Expect.*, vol. 21, no. 6, pp. 939–943, 2018, doi: 10.1111/hex.12800.

[7] G. A. K. van Voorn *et al.*, "The Missing Stakeholder Group: Why Patients Should be Involved in Health Economic Modelling," *Appl. Health Econ. Health Policy*, vol. 14, no. 2, pp. 129–133, 2016, doi: 10.1007/s40258-015-0200-7.

[8] J. Cane, D. O'Connor, and S. Michie, "Validation of the theoretical framework," *Implement. Sci.*, vol. 7, p. 37, 2012.

[9] J. Presseau *et al.*, "Barriers and facilitators to healthcare professional behaviour change in clinical trials using the Theoretical Domains Framework: A case study of a trial of individualized temperature-reduced haemodialysis," *Trials*, vol. 18, no. 1, pp. 1–16, 2017, doi: 10.1186/s13063-017-1965-9.

[10] W. Skoien, K. Page, W. Parsonage, S. Ashover, T. Milburn, and L. Cullen, "Use of the Theoretical Domains Framework to evaluate factors driving successful implementation of the Accelerated Chest pain Risk Evaluation (ACRE) project," *Implement. Sci.*, vol. 11, no. 1, pp. 1–11, 2016, doi: 10.1186/s13012-016-0500-9.

[11] J. M. Huijg *et al.*, "Measuring determinants of implementation behavior: Psychometric properties of a questionnaire based on the theoretical domains framework," *Implement. Sci.*, vol. 9, no. 1, pp. 1–15, 2014, doi: 10.1186/1748-5908-9-33.

[12] R. Newlands *et al.*, "Barriers and facilitators of evidence-based management of patients with bacterial infections among general dental practitioners: A theory-informed interview study," *Implement. Sci.*, vol. 11, no. 1, pp. 1–12, 2016, doi: 10.1186/s13012-016-0372-z.

[13] M. Z. Raza, H. Bruhn, and K. Gillies, "Dissemination of trial results to participants in phase III pragmatic clinical trials: An audit of trial investigators intentions," *BMJ Open*, vol. 10, no. 1, pp. 1–8, 2020, doi: 10.1136/bmjopen-2019-035730.

[14] K. McKenzie, A., Bulsara, C., Haines, H., Hanley, B., Alpers, "Barriers to community involvement in health and medical research," 2016.

[15] L. Dudley *et al.*, "A little more conversation please? Qualitative study of researchers' and patients' interview accounts of training for patient and public involvement in clinical trials," *Trials*, vol. 16, no. 1, pp. 1–15, 2015, doi: 10.1186/s13063-015-0667-4.

[16] I. Schilling *et al.*, "Patients' and researchers' experiences with a patient board for a clinical trial on urinary tract infections," *Res. Involv. Engagem.*, vol. 5, no. 1, pp. 1–14, 2019, doi: 10.1186/s40900-019-0172-0.

For peer review only - http://bmjopen.bmj.com/site/about/guidelines.xhtml

[17] L. Locock and A. Boaz, “Drawing straight lines along blurred boundaries: Qualitative research, patient and public involvement in medical research, co-production and co-design,” *Evid. Policy*, vol. 15, no. 3, pp. 409–421, 2019, doi: 10.1332/174426419X15552999451313.

[18] A. Kearney *et al.*, “Priorities for methodological research on patient and public involvement in clinical trials: A modified Delphi process,” *Heal. Expect.*, vol. 20, no. 6, pp. 1401–1410, 2017, doi: 10.1111/hex.12583.

[19] V. Braun and V. Clarke, *Succesful qualitative research*. SAGE, 2013.

For peer review only

For peer review only - http://bmjopen.bmj.com/site/about/guidelines.xhtml

Table 1 – Descriptive statistics by trialists current practice (involving PPP in numerical aspects of trials or not / not sure) and in total – values are n(%)

	Trialists that involve PPP in numerical aspects (N=116)	Trialists that do not involve PPP in numerical aspects or don't know (N=71)	Total
**Age**			
18-24 years old	1 (0.9)	1 (1.4)	2 (1.1)
25-34 years old	32 (27.6)	30 (42.3)	62 (33.2)
35-44 years old	38 (32.8)	12 (16.9)	50 (26.7)
45-54 years old	25 (21.6)	16 (22.5)	41 (21.9)
55-64 years old	14 (12.1)	8 (11.3)	22 (11.8)
65+ years old	5 (4.3)	2 (2.8)	7 (3.7)
Rather not say	1 (0.9)	2 (2.8)	3 (1.6)
**Gender**			
Female	80 (69.0)	50 (70.4)	130 (69.5)
Male	33 (28.4)	17 (23.9)	50 (26.7)
Other		2 (2.8)	2 (1.1)
Prefer not to say	3 (2.6)	2 (2.8)	5 (2.7)
**Work location**			
England	9 (7.8)	8 (11.3)	17 (9.1)
North East England	4 (3.4)	3 (4.2)	7 (3.7)
North West England	7 (6.0)	7 (9.9)	14 (7.5)
Yorkshire and the Humber	8 (6.9)	5 (7.0)	13 (7.0)
West Midlands	10 (8.6)	2 (2.8)	12 (6.4)
East Midlands	3 (2.6)	4 (5.6)	7 (3.7)
South West England	9 (7.8)	2 (2.8)	11 (5.9)
South East England	13 (11.2)	6 (8.5)	19 (10.2)
London	19 (16.4)	14 (19.7)	33 (17.6)
East England	3 (2.6)	1 (1.4)	4 (2.1)
Northern Ireland	3 (2.6)	3 (4.2)	6 (3.2)
Scotland	18 (15.5)	12 (16.9)	30 (16.0)
Wales	10 (8.6)	4 (5.6)	14 (7.5)
**Which aspects of the trial process are you directly involved in?**			
Trial design	51 (44.0)	37 (52.1)	88 (47.1)
Undertaking the trial	48 (41.4)	37 (52.1)	85 (45.5)
Dissemination	55 (47.4)	34 (47.9)	89 (47.6)
Analysis	36 (31.0)	23 (32.4)	59 (31.6)
Overseeing committee	1 (0.9)	4 (5.6)	5 (2.7)
All stages	56 (48.3)	19 (26.8)	75 (40.1)
**Main role in trial**			
Trial manager	81 (69.8)	44 (62.0)	125 (66.8)
Chief investigator	2 (1.7)	1 (1.4)	3 (1.6)
Statistician	16 (13.8)	13 (18.3)	29 (15.5)
Health economist	2 (1.7)	1 (1.4)	3 (1.6)

For peer review only - http://bmjopen.bmj.com/site/about/guidelines.xhtml

For peer review only

Qualitative researcher	6 (5.2)	2 (2.8)	8 (4.3)
Patient Partner	4 (3.4)	4 (5.6)	8 (4.3)
Co-investigator	9 (7.8)	7 (9.9)	16 (8.6)
Researcher	6 (5.2)	1 (1.4)	7 (3.7)
Systematic reviewer		1 (1.4)	1 (0.5)
PPI lead	1 (0.9)	2 (2.8)	3 (1.6)
Not reported	1 (0.9)		1 (0.5)
**How long have you been working in trials?**			
Less than 5 years	28 (24.1)	29 (40.8)	57 (30.5)
Between 5-10 years	44 (37.9)	17 (23.9)	61 (32.6)
More than 10 years	44 (37.9)	25 (35.2)	69 (36.9)
**When do you involve PPP in numerical aspects of trials?**			
Never	2 (1.7)		2 (1.1)
Rarely	10 (8.6)		10 (5.3)
Regularly	72 (62.1)		72 (38.5)
Every day	32 (27.6)		32 (17.1)
**What stage of a trial do you involve PPP in the numerical aspects?**			
Trial design	86 (74.1)		86 (46.0)
Undertaking the trial	58 (50.0)		58 (31.0)
Dissemination	84 (72.4)		84 (44.9)
Undertaking the trial	58 (50.0)		58 (31.0)
Overseeing committee	8 (6.9)		8 (4.3)
No information available	2 (0.9)		4 (1.6)

For peer review only

Table – Responses to selected Theoretical Domains Framework (TDF) questions by whether respondent said they involved patient and public partners (PPP) in numerical aspects of trials – median (percentile 25 – percentile 75), count for scale variables or n (%) out of N for binary variables

	Respondents involve PPP (N=116)	Respondents do not involve PPP or are not sure (N=71)
**TDF domain:** Knowledge		
How familiar are you with involving PPP in numerical aspects of trials?	4 (3.0-5.0),116	2 (1.0-3.0),71
**TDF domain:** Skills		
Grade your own ability to involve PPP in numerical aspects of trials	4 (3.0-5.0),116	2 (1.0-4.0),71
**PPP skills**		
**Do you think the PPP need to have particular experience or skills to be involved in numerical aspects of trials?**		
Yes	52 (44.8)	41 (57.7)
No	64 (55.2)	30 (42.3)
**TDF domain:** Social / professional role		
Is involving PPP in numerical aspects of trials an expected role within your job?	46 (39.7)	3 (4.2)
**TDF domain:** Beliefs about capability		
Do you feel confident in your ability to involve PPP in numerical aspects of trials?	4 (3.0-5.0),111	3 (2.0-4.0),62
Do you think that involving PPP in numerical aspects of trials is hard to deliver?	52 (44.8)	34 (47.9)
**TDF domain:** Beliefs about consequences		
There is a good balance between the challenges of involving PPP in numerical aspects of trials and the potential benefits	68 (58.6)	22 (31.0)
**TDF domain:** Reinforcement		
You get recognition from PPP, when you involve them in numerical aspects of trials	35 (30.2)	12 (16.9)
You get recognition from work peers	21 (18.1)	5 (7.0)
You get recognition from your manager	23 (19.8)	6 (8.5)
**TDF domain:** Environmental context and resources		
Have the resources needed to involve PPP in numerical aspects of trials	55 (47.4)	16 (22.5)
Employer provides support to involve PPP in numerical aspects of trials	47 (40.5)	13 (18.3)
Employer provides training to involve PPP in numerical aspects of trials	31 (26.7)	7 (9.9)
Involving PPP in numerical aspects of trials is compatible with daily practice	63 (54.3)	17 (23.9)
PPP are motivated to get involved in numerical aspects of trials	59 (50.9)	14 (19.7)
**TDF domain:** Social influences		
Most people who are important think I should involve PPP in numerical aspects of trials	5 (4.0-6.0),94	3 (1.0-4.0),50

For peer review only

For peer review only - http://bmjopen.bmj.com/site/about/guidelines.xhtml

**Who encourages you to involve PPP in numerical aspects?**		
No encouragement	7 (6.0)	14 (19.7)
**Who is a barrier for you to involve PPP in numerical aspects?**		
No barrier	47 (40.5)	13 (18.3)
**TDF domain:** Behavioural regulation		
**Do you have a clear plan on…**		
Which numerical aspects of trials you should involve PPP with?	33 (28.4)	5 (7.0)
How you will involve PPP in numerical aspects of trials?	36 (31.0)	6 (8.5)
How often will you involve PPP in numerical aspects of trials?	37 (31.9)	4 (5.6)

For peer review only

For peer review only - http://bmjopen.bmj.com/site/about/guidelines.xhtml

Table 3 – Open question responses coded by domain and grouped according to specific beliefs

Domains	Specific belief	Illustrating quotes
Knowledge	Lack of clarity about what the task entails	*"[What would help me involve PPP in numerical aspects of trials is…] Understanding what involving patients and public in numerical aspects of trials actually means, how it can be done and why it's important" [TM, NI]*<br><br>*"I think the main issue here is that there are very simple parts of numerical aspects of trials which we would always, and easily, involve PPI in: outcome measure acceptability and monitoring trial progress. I don't know what other aspects might be, so I am imagining a training or knowledge failure on my part." [TM, involves]*
	Training	*"[What would help me involve PPP in numerical aspects is…] training courses/workshops for members of the public on the numerical aspects of trials and also researchers on how to involve them" [HE, NI]*<br><br>*"This is something that has never really been discussed at work. Nor have I seen any courses about doing this. More guidance is available about writing simply for patients but not specifically about numerical aspects. Some training would be welcome." [TM, involves]*
	Knowledge of patient and public partners	*"[What makes involving PPP in numerical aspects hard is…] Being confident patients have sufficient understanding to be able to contribute. Not sure what could make it easier, just need to be aware this may be a potential issue during initial discussions with focus group members for example." [TM, involves]*<br><br>*"[What makes involving PPP in numerical aspects hard is…] a general lack of awareness of how trials (and research) work, which makes the initial engagement harder. It's perfectly possible to overcome this though." [Qualitative researcher, involves]*

For peer review only - http://bmjopen.bmj.com/site/about/guidelines.xhtml

Skills	Skills of patient and public partners	*"… I would suggest that if you want to involve PPI in this [numerical] aspect then you would have to purposively approach PPI with a certain skill set or advertise for such PPI involvement" [Co-investigator, NI]*<br><br>*"It generally quite boring for the general public and they do need a basic level of numeracy and appreciation of research methods" [Statistician, involves]*
Social / professional role	Professional role	*"The grant applicants and senior team often take these decisions during trial design. I never had any impact on these decisions previously." [Qualitative researcher, involves]*<br><br>*"Numerical roles already 'assigned' to statisticians and clinicians, so as Trial Manager, it can be difficult to enforce PPI involvement" [TM, involves]*
Beliefs about capability	Capabilities of patient and public partners	*"Often numerical aspects are dictated by statistical programs and I think there is a general feeling that patient or public partners cannot grasp them." [TM, NI]*<br><br>*"[A barrier to involve PPP in numerical aspects of trials is…] The PP themselves not being confident enough to comment" [Co-investigator, involves]*
Beliefs about consequences	Impact on trial quality	*"Patients can help you work out how to disseminate the results e.g. which graphs are easiest, which format of presenting an estimate (risk, number of days etc)" [Statistician, involves]*<br><br>*"Discussing numerical information to patients helps to distill complex findings into something more simple and acceptable. This helps the researcher to clearer think through what are the results, helps explain information clearly that improves the quality of the trial and will ultimately all benefit the patient." [TM, involves]*

For peer review only - http://bmjopen.bmj.com/site/about/guidelines.xhtml

	Positive impact on patient and public partners	*"The PPP can … increase their knowledge and understanding of their condition, new potential treatments, and the process involved in trials which evaluate treatments. This could reduce the information asymmetry between the patient and clinician in the future" [Health Economist, involves]*<br><br>*"Patients can understand the difficulties in running clinical research and trials. Showing recruitment figures and follow-up data is also beneficial as patients can see the results accumulating and the benefits of taking part in trials." [TM, involves]*
	Negative impact on patient and public partners	*"Makes participation stressful, unless the PP has pre-existing skills in stats or maths." [Co-investigator, involves]*
	Extra work / responsibilities for the researcher	*"It is more work for the researcher to involve patients/partners" [Statistician, NI]*
	Uncertainty about impact on trial quality	*"I think it could improve transparency of decision making through a trial process. I am less sure that will actually translate into "better" decisions" [CI, involves]*<br><br>*"I have answered no impact because I think it is unclear whether quality or the patient partner will get a positive impact. That is a question that needs investigation." [CI, involves]*
Reinforcement	Lack of incentives	*"Having some tangible reward for your investment! It never does anything for your career, while other priorities do." [Statistician, involves]*<br><br>*"It's not really rewarded or recognised because it is an expected part of study design. All funders we work with consider PPI involvement with trials in general essential." [TM & statistician, involves]*
Environmental context and resources	Funding	*"[What would help me involve PPP in numerical aspects is…] More funding for a PPI expert at our CTU. I am only funded 10% to be the coordinator for all PPI and to do a good job, I'd like to be able to spend more time on my PPI role" [TM, involves]*
	Time	*"I will need training to effectively include the public and the public will need training to effectively contribute to the research. This will take more time*

For peer review only - http://bmjopen.bmj.com/site/about/guidelines.xhtml

		and resources that aren't currently provided but if they were this would make it much easier" [HE, NI]
	Staff	*"[What would help me involve PPP in numerical aspects is…] More money, more trained help. The usual problems." [TM, involves]*<br><br>*"[What would help me involve PPP in numerical aspects is…]* Having a special person dedicated to running PPI work across trials." *[TM, involves]*
	Difficulty accessing willing volunteers	*"It's difficult to find public partners for any research, but when it's something that's specifically numerical I think that's an even bigger challenge!" [Qualitative researcher & TM, involves]*<br><br>*"[What would help me involve PPP in numerical aspects is…] To know how or where to find participants that would be interested in participate." [TM, NI]*
Social influences	Supportive team environment	*"[What would help me involve PPP in numerical aspects is…] More of a group/team effort so that it is not just left to the trial manager to present to PPI members but the trial team as a whole takes on this responsibility." [TM, involves]*<br><br>*"I think the enthusiasm of the chief investigator to include PPI members is important. If the investigator wants to involve PPI then it will happen but if the investigator is not bothered then it proves much more difficult to include someone." [TM, involves]*
	Patient and public partners' interest	*"I would be happy to share details of the numerical analysis, but have never been asked to do so - they seem satisfied with the summary explanations provided." [TM, NI]*<br><br>*"PPs generally shrink back even when asked to comment on numerical aspects of a trial" [Co-investigator, involves]*

For peer review only - http://bmjopen.bmj.com/site/about/guidelines.xhtml

Behavioural regulation	Guidance	*"… If there was clear guidance and tools on how to do it and those tools could be easily integrated into management structures for running large scale studies then I would use them" [CI, involves]*<br><br>*"[What would help me involve PPP in numerical aspects is…] Guidelines about PPI expectations for review of numerical aspects" [TM, unsure about involvement]*
	Standard Operating Procedures	*"[What would help me involve PPP in numerical aspects is…] have this implemented into SOPs [Standard Operating Procedures]" [TM, NI]*
Trial communication culture	Lack of clarity in communication about numerical and statistical aspects of trials	*"language and jargon used around sample size calculations etc in design discussions. Stats/methodologists need to be open and able to discuss complex issues openly and simply" [TM, involves]*<br><br>*"As a patient partner I feel very privileged ... But as a retired maths teacher, I am very aware of the anxiety that numbers cause even otherwise very clever people - these anxieties will only be mitigated by more involvement at all levels, including the public." [PPP, involves]*<br><br>*"If everyone in the research environment explained what the numbers mean clearly to all members of the team - that would make it easier to describe and facilitate involvement from patient or the public" [TM, NI]*

CI – Chief Investigator; HE – Health Economist; TM – Trial manager; NI – doesn't involve PPP in numerical aspects

For peer review only

Flow diagram of respondents to the survey

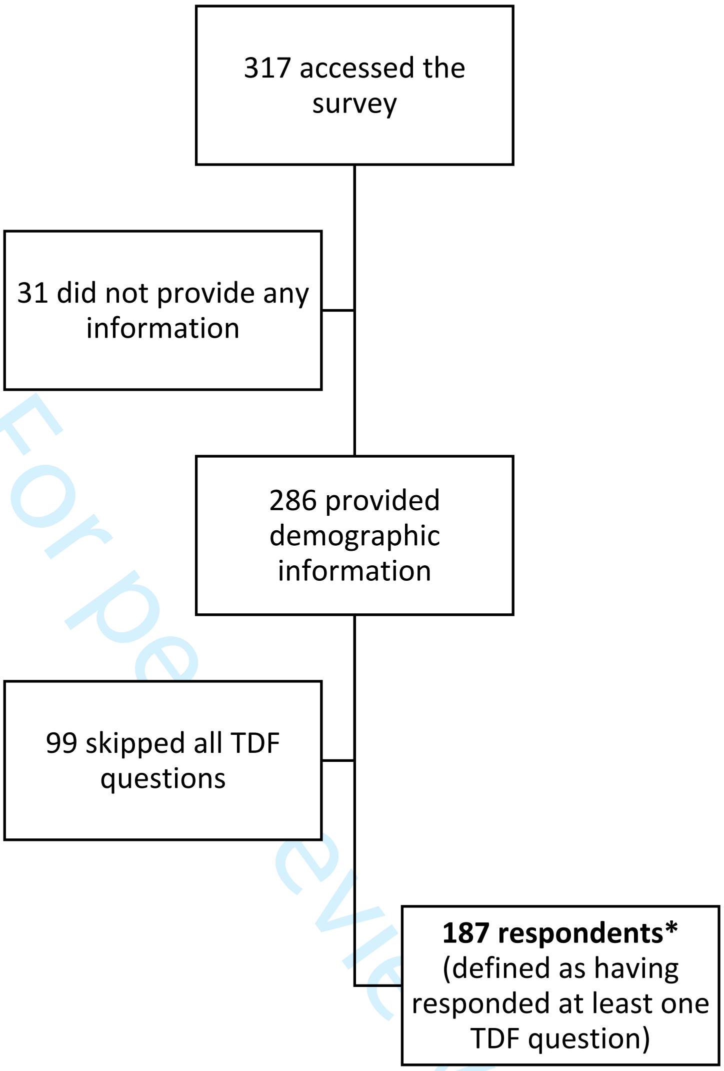

Figure 1 – Flow of participants (* we present demographic information by respondent status in Supplementary Table 1). TDF – Theoretical Domains Framework

For peer review only

Supplementary Table 1 – Demographic characteristics by respondent status (respondents were defined as participants that replied to demographic characteristics and all theoretical domains framework questions; non-respondents were those that replied to demographic questions only)

	Respondents (N=187)	Non-respondents (N=99)
**Age**		
18-24 years old	2 (1.1)	3 (3.0)
25-34 years old	62 (33.2)	27 (27.3)
35-44 years old	50 (26.7)	35 (35.4)
45-54 years old	41 (21.9)	20 (20.2)
55-64 years old	22 (11.8)	9 (9.1)
65+ years old	7 (3.7)	4 (4.0)
Rather not say	3 (1.6)	1 (1.0)
**Gender**		
Female	130 (69.5)	82 (82.8)
Male	50 (26.7)	17 (17.2)
Other	2 (1.1)	
Prefer not to say	5 (2.7)	
**Work location**		
England	17 (9.1)	10 (10.1)
North East England	7 (3.7)	3 (3.0)
North West England	14 (7.5)	7 (7.1)
Yorkshire and the Humber	13 (7.0)	11 (11.1)
West Midlands	12 (6.4)	1 (1.0)
East Midlands	7 (3.7)	6 (6.1)
South West England	11 (5.9)	6 (6.1)
South East England	19 (10.2)	10 (10.1)
London	33 (17.6)	18 (18.2)
East England	4 (2.1)	3 (3.0)
Northern Ireland	6 (3.2)	5 (5.1)
Scotland	30 (16.0)	16 (16.2)
Wales	14 (7.5)	3 (3.0)
**Which aspects of the trial process are you directly involved in?**		
Trial design	88 (47.1)	51 (51.5)
Undertaking the trial	85 (45.5)	50 (50.5)
Dissemination	89 (47.6)	39 (39.4)
Analysis	59 (31.6)	24 (24.2)
Overseeing committee	5 (2.7)	1 (1.0)
All stages	75 (40.1)	25 (25.3)
None		1 (1.0)
**Main role in trial**		
Trial manager	125 (66.8)	69 (69.7)
Chief investigator	3 (1.6)	
Statistician	29 (15.5)	8 (8.1)
Health economist	3 (1.6)	3 (3.0)
Qualitative researcher	8 (4.3)	
Patient Partner	8 (4.3)	3 (3.0)
Co-investigator	16 (8.6)	10 (10.1)

For peer review only - http://bmjopen.bmj.com/site/about/guidelines.xhtml

Researcher	7 (3.7)	6 (6.1)
Systematic reviewer	1 (0.5)	
Programmer		1 (1.0)
PPI lead	3 (1.6)	4 (4.0)
Don't work in a trial		2 (2.0)
Missing	63 (33.2)	30 (30.3)
**How long have you been working in trials?**		
Less than 5 years	57 (30.5)	35 (35.4)
Between 5-10 years	61 (32.6)	28 (28.3)
More than 10 years	69 (36.9)	36 (36.4)
**When do you involve patient or public partners in numerical aspects of trials?**		
Never	7 (3.7)	7 (7.1)
Rarely	23 (12.3)	18 (18.2)
Regularly	109 (58.3)	57 (57.6)
Every day	48 (25.7)	11 (11.1)
Missing		6 (6.1)
**Do you involve patient or public partners in the numerical aspects of trials?**		
Yes	116 (62.0)	18 (18.2)
No	48 (25.7)	6 (6.1)
Don't know	23 (12.3)	3 (3.0)
Missing		72 (72.7)

For peer review only

For peer review only - http://bmjopen.bmj.com/site/about/guidelines.xhtml

Supplementary Table 2 – Responses to the Theoretical Domains Framework questions by whether respondent said they involved patient and public partners (PPP) in numerical aspects of trials – median (percentile 25 – percentile 75), count for scale variables or n (%) out of N for binary variables

	Respondents involve PPP (N=116)	Respondents do not involve PPP or are not sure (N=71)
**TDF domain:** Knowledge		
How familiar are you with involving PPP in numerical aspects of trials?	4 (3.0-5.0),116	2 (1.0-3.0),71
**TDF domain:** Skills		
Grade your own ability to involve PPP in numerical aspects of trials	4 (3.0-5.0),116	2 (1.0-4.0),71
**TDF domain:** Social / professional role		
Is involving PPP in numerical aspects of trials an expected role within your job?	46 (39.7)	3 (4.2)
**TDF domain: Beliefs about capability**		
Do you feel confident in your ability to involve PPP in numerical aspects of trials?	4 (3.0-5.0),111	3 (2.0-4.0),62
Do you think that involving PPP in numerical aspects of trials is hard to deliver?	52 (44.8)	34 (47.9)
**TDF domain:** Optimism		
Involving PPP in numerical aspects of trials is a good thing	6 (5.0-7.0),108	5 (4.0-6.0),61
**Do you think involving patient or partners in numerical aspects of a trial can have a positive impact on the researcher?**		
Yes	96 (82.8)	43 (60.6)
No impact	5 (4.3)	11 (15.5)
Negative impact	1 (0.9)	2 (2.8)
Missing	14 (12.1)	15 (21.1)
**on the quality of the trial?**		
Yes	93 (80.2)	46 (64.8)
No impact	8 (6.9)	9 (12.7)
Negative impact	1 (0.9)	1 (1.4)
Missing	14 (12.1)	15 (21.1)
**on the patient partner?**		
Yes	95 (81.9)	50 (70.4)
No impact	6 (5.2)	4 (5.6)
Negative impact	1 (0.9)	2 (2.8)
Missing	14 (12.1)	15 (21.1)
**TDF domain:** beliefs about consequences		
There is a good balance between the challenges of involving PPP in numerical aspects of trials and the potential benefits	68 (58.6)	22 (31.0)
**TDF domain:** Reinforcement		
You get recognition from PPP, when you involve them in numerical aspects of trials	35 (30.2)	12 (16.9)
You get recognition from work peers	21 (18.1)	5 (7.0)

For peer review only - http://bmjopen.bmj.com/site/about/guidelines.xhtml

You get recognition from your manager	23 (19.8)	6 (8.5)
**TDF domain:** Intention		
**Will you involve PPP in numerical aspects of your next project?**		
Yes definitely	41 (35.3)	4 (5.6)
Yes probably	42 (36.2)	16 (22.5)
No definitely	0	1 (1.4)
No probably	4 (3.4)	9 (12.7)
I don't know	12 (10.3)	24 (33.8)
Missing	17 (14.7)	17 (23.9)
**TDF domain:** Goals		
Working on something else on my agenda is a higher priority than involving PPP in the numerical aspects of trials	4 (3.0-5.0),99	5 (4.0-6.0),54
**TDF domain:** Memory, attention, decision process		
Involving PPP in numerical aspects of trials is something I do automatically	4 (3.0-6.0),93	2 (1.0-2.5),52
**TDF domain:** Environmental context and resources		
Have the resources needed to involve PPP in numerical aspects of trials	55 (47.4)	16 (22.5)
Employer provides support to involve PPP in numerical aspects of trials	47 (40.5)	13 (18.3)
Employer provides training to involve PPP in numerical aspects of trials	31 (26.7)	7 (9.9)
Involving PPP in numerical aspects of trials is compatible with daily practice	63 (54.3)	17 (23.9)
PPP are motivated to get involved in numerical aspects of trials	59 (50.9)	14 (19.7)
**TDF domain:** Social influences		
Most people who are important think I should involve PPP in numerical aspects of trials	5 (4.0-6.0),94	3 (1.0-4.0),50
**Who encourages you to involve PPP in numerical aspects?**		
Myself	63 (54.3)	15 (21.1)
Co-workers	45 (38.8)	11 (15.5)
Manager	44 (37.9)	10 (14.1)
Social media	14 (12.1)	6 (8.5)
My personal environment	18 (15.5)	1 (1.4)
Scientific literature	31 (26.7)	15 (21.1)
Public	24 (20.7)	9 (12.7)
Institution	38 (32.8)	10 (14.1)
Regulation	22 (19.0)	15 (21.1)
Mass media	3 (2.6)	3 (4.2)
No encouragement	7 (6.0)	14 (19.7)
**Who is a barrier for you to involve PPP in numerical aspects?**		
Myself	6 (5.2)	7 (9.9)
Co-workers	4 (3.4)	7 (9.9)

For peer review only - http://bmjopen.bmj.com/site/about/guidelines.xhtml

Manager	6 (5.2)	10 (14.1)
Scientific literature	2 (1.7)	3 (4.2)
Social media	2 (1.7)	
My personal environment	11 (9.5)	6 (8.5)
Public	5 (4.3)	7 (9.9)
Institution	13 (11.2)	13 (18.3)
Regulation	10 (8.6)	5 (7.0)
Mass media	5 (4.3)	3 (4.2)
No barrier	47 (40.5)	13 (18.3)
**TDF domain:** Emotions		
**Emotions related to involving PPP in numerical aspects**		
Optimism	65 (56.0)	29 (40.8)
Ease	18 (15.5)	5 (7.0)
Calm	14 (12.1)	3 (4.2)
Relaxation	3 (2.6)	3 (4.2)
Appreciation	71 (61.2)	32 (45.1)
Nervousness	37 (31.9)	18 (25.4)
Pessimism	7 (6.0)	12 (16.9)
Depression		2 (2.8)
Agitation	9 (7.8)	11 (15.5)
Happiness	27 (23.3)	8 (11.3)
Sadness	2 (1.7)	2 (2.8)
Anxiety	25 (21.6)	17 (23.9)
**TDF domain:** Behavioural regulation		
**Do you have a clear plan on…**		
Which numerical aspects of trials you should involve PPP with?	33 (28.4)	5 (7.0)
How you will involve PPP in numerical aspects of trials?	36 (31.0)	6 (8.5)
How often will you involve PPP in numerical aspects of trials?	37 (31.9)	4 (5.6)
**TDF domain:** Nature of behaviour		
Involve PPP in numerical aspects of trials without consciously thinking about it	4 (2.0-5.0),90	2 (1.0-3.5),48

For peer review only - http://bmjopen.bmj.com/site/about/guidelines.xhtml

Reporting checklist for cross sectional study.

Based on the STROBE cross sectional guidelines.

Instructions to authors

Complete this checklist by entering the page numbers from your manuscript where readers will find each of the items listed below.

Your article may not currently address all the items on the checklist. Please modify your text to include the missing information. If you are certain that an item does not apply, please write "n/a" and provide a short explanation.

Upload your completed checklist as an extra file when you submit to a journal.

In your methods section, say that you used the STROBE cross sectionalreporting guidelines, and cite them as:

von Elm E, Altman DG, Egger M, Pocock SJ, Gotzsche PC, Vandenbroucke JP. The Strengthening the Reporting of Observational Studies in Epidemiology (STROBE) Statement: guidelines for reporting observational studies.

For peer review only

		Reporting Item	Page Number
**Title and abstract**			
Title	#1a	Indicate the study’s design with a commonly used term in the title or the abstract	1
Abstract	#1b	Provide in the abstract an informative and balanced summary of what was done and what was found	1
**Introduction**			
Background / rationale	#2	Explain the scientific background and rationale for the investigation being reported	3
Objectives	#3	State specific objectives, including any prespecified hypotheses	3
**Methods**			
Study design	#4	Present key elements of study design early in the paper	3
Setting	#5	Describe the setting, locations, and relevant dates, including periods of	4

		recruitment, exposure, follow-up, and data collection	
Eligibility criteria	#6a	Give the eligibility criteria, and the sources and methods of selection of participants.	4
	#7	Clearly define all outcomes, exposures, predictors, potential confounders, and effect modifiers. Give diagnostic criteria, if applicable	3-4
Data sources / measurement	#8	For each variable of interest give sources of data and details of methods of assessment (measurement). Describe comparability of assessment methods if there is more than one group. Give information separately for for exposed and unexposed groups if applicable.	3-4
Bias	#9	Describe any efforts to address potential sources of bias	NA
Study size	#10	Explain how the study size was arrived at	3-4
Quantitative variables	#11	Explain how quantitative variables were handled in the analyses. If applicable, describe which groupings were chosen, and why	4-5
Statistical methods	#12a	Describe all statistical methods, including those used to control for confounding	4-5
Statistical methods	#12b	Describe any methods used to examine subgroups and interactions	4-5
Statistical methods	#12c	Explain how missing data were addressed	4-5
Statistical methods	#12d	If applicable, describe analytical methods taking account of sampling strategy	4-5
Statistical methods	#12e	Describe any sensitivity analyses	4-5
**Results**			
Participants	#13a	Report numbers of individuals at each stage of study—eg numbers potentially eligible, examined for eligibility, confirmed eligible, included in the study, completing follow-up, and analysed. Give information separately for for exposed and unexposed groups if applicable.	5, Fig 1
Participants	#13b	Give reasons for non-participation at each stage	NA
Participants	#13c	Consider use of a flow diagram	5, Fig 1

For peer review only

Descriptive data	#14a	Give characteristics of study participants (eg demographic, clinical, social) and information on exposures and potential confounders. Give information separately for exposed and unexposed groups if applicable.	p5, Table 1
Descriptive data	#14b	Indicate number of participants with missing data for each variable of interest	p5, Tables 1,2
Outcome data	#15	Report numbers of outcome events or summary measures. Give information separately for exposed and unexposed groups if applicable.	Table 2
Main results	#16a	Give unadjusted estimates and, if applicable, confounder-adjusted estimates and their precision (eg, 95% confidence interval). Make clear which confounders were adjusted for and why they were included	NA
Main results	#16b	Report category boundaries when continuous variables were categorized	NA
Main results	#16c	If relevant, consider translating estimates of relative risk into absolute risk for a meaningful time period	NA
Other analyses	#17	Report other analyses done—e.g., analyses of subgroups and interactions, and sensitivity analyses	NA
**Discussion**			
Key results	#18	Summarise key results with reference to study objectives	9-11
Limitations	#19	Discuss limitations of the study, taking into account sources of potential bias or imprecision. Discuss both direction and magnitude of any potential bias.	11
Interpretation	#20	Give a cautious overall interpretation considering objectives, limitations, multiplicity of analyses, results from similar studies, and other relevant evidence.	9-11
Generalisability	#21	Discuss the generalisability (external validity) of the study results	9-11
**Other Information**			
Funding	#22	Give the source of funding and the role of the funders for the present study and, if applicable, for the original study on which the present article is based	12

Notes:

For peer review only

- 14a: p5, Table 1
- 14b: p5, Tables 1,2 The STROBE checklist is distributed under the terms of the Creative Commons Attribution License CC-BY. This checklist was completed on 14. November 2020 using https://www.goodreports.org/, a tool made by the EQUATOR Network in collaboration with Penelope.ai

For peer review only

BMJ Open

Patient and public involvement in numerical aspects of trials: a mixed methods theory-informed survey of trialists' current practices, barriers and facilitators

Journal:	*BMJ Open*
Manuscript ID	bmjopen-2020-046977.R1
Article Type:	Original research
Date Submitted by the Author:	01-Feb-2021
Complete List of Authors:	Goulao, Beatriz; University of Aberdeen, Health Services Research Unit<br>Poisson, Camille; University of Aberdeen, Health Services Research Unit<br>Gillies, Katie; University of Aberdeen, Health Services Research Unit
**Primary Subject Heading**:	Health services research
Secondary Subject Heading:	Health services research
Keywords:	Clinical trials < THERAPEUTICS, STATISTICS & RESEARCH METHODS, HEALTH SERVICES ADMINISTRATION & MANAGEMENT

SCHOLARONE™
Manuscripts

I, the Submitting Author has the right to grant and does grant on behalf of all authors of the Work (as defined in the below author licence), an exclusive licence and/or a non-exclusive licence for contributions from authors who are: i) UK Crown employees; ii) where BMJ has agreed a CC-BY licence shall apply, and/or iii) in accordance with the terms applicable for US Federal Government officers or employees acting as part of their official duties; on a worldwide, perpetual, irrevocable, royalty-free basis to BMJ Publishing Group Ltd ("BMJ") its licensees and where the relevant Journal is co-owned by BMJ to the co-owners of the Journal, to publish the Work in this journal and any other BMJ products and to exploit all rights, as set out in our licence.

The Submitting Author accepts and understands that any supply made under these terms is made by BMJ to the Submitting Author unless you are acting as an employee on behalf of your employer or a postgraduate student of an affiliated institution which is paying any applicable article publishing charge ("APC") for Open Access articles. Where the Submitting Author wishes to make the Work available on an Open Access basis (and intends to pay the relevant APC), the terms of reuse of such Open Access shall be governed by a Creative Commons licence – details of these licences and which Creative Commons licence will apply to this Work are set out in our licence referred to above.

Other than as permitted in any relevant BMJ Author's Self Archiving Policies, I confirm this Work has not been accepted for publication elsewhere, is not being considered for publication elsewhere and does not duplicate material already published. I confirm all authors consent to publication of this Work and authorise the granting of this licence.

For peer review only

Patient and public involvement in numerical aspects of trials: a mixed methods theory-informed survey of trialists' current practices, barriers and facilitators

Beatriz Goulao[1], beatriz.goulao@abdn.ac.uk, corresponding author

Camille Poisson[1], hsru.aberdeen@abdn.ac.uk

Katie Gillies[1], k.gilles@abdn.ac.uk

1 – Health Services Research Unit, University of Aberdeen

Keywords: Patient and public involvement; clinical trials; trial methodology; theoretical domains framework

Abstract (228/300)

Objectives: We aimed to find out if trialists involve patients and the public in numerical aspects of trials, how and what are the barriers and facilitators to doing it.

Design: We developed a survey based on the theoretical domains framework. We used a mixed methods approach to analyse the data and to identify important domains.

Setting: Online survey targeting UK-based trial units

Participants: Stakeholders working in UK-based clinical trials, 18 years old or over, to understand English and to agree to take part in the study.

Outcome measures: Trialists' behaviour of involving patients and the public in numerical aspects of trials and its determinants

Results: We included 187 responders. The majority were female (70%), trial managers (67%) and involved public and patient partners in numerical aspects of trials (60%). We found lack of knowledge, trialists' perception of public and patient partners' skills, capabilities and motivations, scarce resources, lack of reinforcement, and lack of guidance were barriers to involving public and patient partners in numerical aspects of trials. Positive beliefs about consequences were an incentive to doing it.

Conclusions: More training, guidance and funding can help trialists involve patient and public partners in numerical aspects, although they were uncertain about public and patient partners' motivation to be involved. Future research should focus on identifying public and patient partners' motivations, and develop strategies to improve the communication of numerical aspects.

For peer review only - http://bmjopen.bmj.com/site/about/guidelines.xhtml
For peer review only

Summary of the article

Strengths and limitations of this study

- First investigation of trialists practice regarding involving patients and the public in numerical aspects of trials and aimed at UK based trialists
- We achieved a wide representation of trialists working in clinical trials in the UK
- We describe current practice as well as determinants of behaviour using a theory-informed approach based on the theoretical domains framework
- Respondents' interpretation of involving patients and the public numerical aspects varied, so the results apply to a spectrum of behaviours. Respondents are likely to have stronger views of patient and public involvement (PPI) and PPI in numerical aspects of trial since they opted to answer our survey.

For peer review only

For peer review only - http://bmjopen.bmj.com/site/about/guidelines.xhtml

Background

Patient and Public Involvement (PPI) has been defined as “research being carried out ‘with’ or ‘by’ members of the public (including patients and carers) rather than ‘to’, ‘about’ or ‘for’ them.” (http://www.invo.org.uk/) PPI activity has become increasingly common in trials in the United Kingdom (UK) for several reasons: its supporters believe it makes research more relevant and it is, morally, the right thing to do. It is also a requirement from major funders, including the National Institute for Health Research (NIHR).[1]

In a trial context, patient and public partners have been primarily involved in agenda setting, steering committees, ethical review, protocol development, and piloting [2] but PPI remains seen as tokenistic and a ticking box exercise in many settings [3], [4]. Trials are primarily quantitative research and often key decisions are based on numerical aspects and their discussion. However, the extent to which patients and the public get involved in those discussions is unknown with some evidence suggesting communication of statistical aspects of trials needs to be clearer in order to achieve meaningful involvement [5]. At the same time, involving patients and the public in statistical and health economics aspects of trials can result in enriching data interpretation, better research, with more robust and implementable evidence [6], [7].

Patient and public involvement can be considered a professional behavioural since it involves a series of actions to accomplish a goal (such as inviting a patient partner to contribute to a discussion). To understand a specific behaviour and its determinants it can be helpful to use behavioural theory to provide a systematic and replicable framework. The Theoretical Domains Framework (TDF) was developed to focus on healthcare professional behaviour, specifically around evidence-based medicine and the adoption of new practices and is made of up of 15 behavioural domains [8]. Previous work has used the TDF to explore researchers’ beliefs related to PPI in health research (Boylan), but to our knowledge this is the first application of the framework to explore PPI in trials and, in particular, PPI in numerical aspects of trials. We aimed to use a theory-informed approach to find out whether trialists involve patient and public partners in numerical aspects of trials and what are the barriers and facilitators to doing it.

Methods

We conducted an online survey approved the College of Ethics Research Committee (CERB/2019/4/1759) of the University of Aberdeen.

Survey design

The survey was divided into the following sections:

- Demographic questions
- Definition of numerical aspects of trials
- General questions about PPI involvement in numerical aspects of trials
- Theoretical domain framework questions

Demographic questions focused on characterising our sample (age, gender, trial work experience and role). We agreed the definition of “Numerical aspects of trials” ("Any aspects of a trial where people measure (or plan to measure), manage, analyse or share information that is presented as one or more numbers.") through an iterative process involving thirteen members of the Health Services Research

For peer review only

For peer review only - http://bmjopen.bmj.com/site/about/guidelines.xhtml

Unit (HSRU) at the University of Aberdeen (including a variety of different roles in the unit, such as a Professor of Health Services Research, a health economist, a statistician, trial manager, PPI/engagement co-ordinator, research fellow, assistant researcher, and quality assurance manager). The definition was included in the survey. Responders were asked about their current practice regarding involving public and patient partners in numerical aspects of trials.

The TDF was developed as a theoretical framework and includes behavioural theories and constructs and proposes that determinants of professionals' behaviour can be clustered in "domains". The TDF, after update from Presseau et al [9], consists of fifteen domains. The TDF section of our survey was developed based on previous surveys [10], [11] and was reviewed by the project team and by an expert in TDF research (ED).

The survey was piloted by eight staff or student members from the University of Aberdeen. The survey was administered and delivered through Survey Monkey. Informed consent was requested and embedded in the survey. The final version contained 43 questions consisting of both closed and open questions (see **Appendix 1 – questionnaire**). The participants were able to withdraw from the survey at any time.

Target population

The target population was any stakeholder working in clinical trials (from here onwards called a "trialist"), for example: trial managers, principal or chief investigators, statisticians, clinicians, patient or public partners, PPI coordinators, research fellows. To take part in our study, the participants needed to be 18 years old or over, to work in a UK clinical trial's unit, to understand English and to agree to take part in the study.

Participant recruitment

The questionnaire was disseminated using an online link via email and social media. We used a snowball approach to recruit survey responders by disseminating the survey to colleagues, known networks, and through social media (LinkedIn, Twitter, Facebook and Instagram). We used common hashtags (#) and pictures to encourage responses. We contacted stakeholders via professional mailing lists to ask to disseminate our survey, such as: PPI coordinator's mailing lists, clinical trial units (CTU) websites and UK Clinical Research Collaboration. The survey link was active from the 11th of June 2019 to the 1st of July 2019.

Survey analysis

The survey analysis was divided in two parts: analysis of the closed-ended questions (as quantitative data) and the analysis of open questions (as qualitative data). We exported the data into Stata and generated statistical summaries of the closed-ended questions. The Likert scale questions used a scale from 1 (weaker agreement / confidence / etc) to 7 (stronger agreement / confidence / etc). They were presented using medians, interquartile ranges and counts. Binary questions (with only two possible answers, for example yes/no) are presented as n (%) out of the available answers. We split results into respondents that self-report to perform the behaviour assessed (involving public and patient partners in numerical aspects of trials) and those that did not or were not sure. When questions included a category named "other", the answers given were grouped together, coded and counted. Analysis of open-ended questions was done using a deductive thematic analysis approach informed by the TDF by one author (BG). If data did not fit into the pre-specified TDF domains, an inductive approach was

For peer review only - http://bmjopen.bmj.com/site/about/guidelines.xhtml

taken. We compared the quantitative and qualitative data to identify the most salient domains of the TDF that explained the behaviour. This process was done by one author (BG) and reviewed using a subset of qualitative data by a second author (KG). We did not undertake any statistical testing since our aim was to describe the data and not to test hypotheses. We used Stata 15 for the purposes of summarising quantitative data and Microsoft Word to analyse qualitative data.

Identifying the most "relevant" domains to involve patients and the public in numerical aspects of trials

In order to establish the barriers and facilitators of the target behaviour (patient and public involvement in numerical aspects of trials), criteria were developed to determine which domains of the TDF were 'relevant' for this behaviour based on previous TDF research [12]. Relevant domains were those that were considered to show variation in reported behaviour/beliefs between trialists that involve patients and the public in numerical aspects and those that don't.

Patient and public involvement in the study

Our study is part of a larger project, the PoINT project (https://www.abdn.ac.uk/hsru/what-we-do/research/projects/point-827.php), where patient and public involvement has been sought at all stages including in its design and via focus groups. The current survey was piloted by three public members.

Results

A total of 317 people accessed the survey. From those 317, we excluded from the final analysis 130 people for the following reasons: 31 did not provide any information; 99 did not respondent to any TDF focussed questions. Therefore, the 187 participants that replied to at least one TDF question were included in the final analysis (**Figure 1**). **Supplementary table 1** provides demographic information based on respondent status. Respondents and non-respondents had similar features with a few exceptions: there was a higher percentage of women in the group of non-respondents compared with respondents (n=82, 83 vs n=130, 70%); there was a higher percentage of respondents involved in all stages of trial (n=75, 40%) compared with non-respondents (n=25, 25%), however the most common trial stages that respondents and non-respondents were involved in were the same: trial design (around 50%) and undertaking the trial (around 50%). There was a higher proportion of statisticians in the respondents' group (n=29, 16%) compared with non-respondents (n=8, 8%). A larger percentage of respondents reported to involve patient and public partners in numerical aspects of trials daily compared with non-respondents (n=48, 26 vs n=11, 11%).

Characteristics of respondents

Table 1 shows characteristics of respondents. Seventy per cent were women (n=130); respondents were based across the UK with the highest percentage coming from London (n=33, 18%). Around 40% were involved in all stages of a trial (n=75) and about half were involved in design; conduct; dissemination. One third were involved in the analysis of a trial. Most respondents were trial managers (n=125, 67%); eight respondents (4%) were patient partners in trials. About 37% (n=69) had been working in trials for more than 10 years and 31% for less than 5 (n=57).

Respondents current practice of involving public and patient partners in numerical aspects of trials

For peer review only - http://bmjopen.bmj.com/site/about/guidelines.xhtml

Around 62% of respondents said they involve public and patient partners in numerical aspects of trials (n=116); 26% said they did not (n=48) and 12% said they were not sure (n=23). From the trialists involving public and patient partners in numerical aspects of trials, 24% were working in the field for less than 5 years (n=28) versus 41% that did not involve public and patient partners in numerical aspects of trials (n=29). Some respondents were uncertain about what involving public and patient partners in numerical aspects meant, with definitions ranging from any discussion about numbers to involvement in the statistical analysis.

The most common stages of trials to involve public and patient partners in numerical aspects were trial design (n=86, 74%) and dissemination (n=84, 72%). About half involved public and patient partners in the analysis and interpretation. Respondents exemplified how they involved public and patient partners in numerical aspects: in management and steering committees where numerical data is discussed; sample size calculations and, specifically, discussing effect sizes, clinically important differences and non-inferiority margins; dissemination of results and aiding to increase clarity of communication with trial participants; grant applications and trial design stage; when and how to collect data; defining outcomes. Comments made about involving public and patient partners in the statistical analysis came from those that didn't see an interest from public and patient partners in getting involved (for example, *"I've also yet to have any PPI members say they would like to be more involved in this side of things; except for post-analysis when considering the qualitative and quantitative aspects together" [Trial manager and statistician, involves]*).

Domains influencing trialists in involving patients and the public in numerical aspects of trials

TDF domains selected as the most relevant for the target behaviour are presented in **Table 2**; all TDF domains results are presented in **Supplementary Table 2**. **Table 3** summarises themes identified from the responses to open questions. By comparing the quantitative and qualitative data, we identified knowledge, skills, beliefs about capabilities, beliefs about consequences, reinforcement, environmental context and resources, social influences and behavioural regulation as being salient TDF domains. We identified an additional theme within the data on "trial communication culture" that did not fit within the TDF domains. Each of the salient TDF domains and the additional theme are presented below.

Knowledge (awareness of involving patients and the public in numerical aspects of trials)

Respondents who involved public and patient partners in numerical aspects scored higher in the knowledge domain (median 4 vs 2 out of 7 (very familiar with PPI in numerical aspects of trials) for those that did and did not involve public and patient partners in numerical aspects, respectively). Knowledge was highlighted as a barrier to involving public and patient partners in numerical aspects of trials in the open questions where respondents discussed their lack of understanding of how or when to involve public and patient partners and suggesting training as a solution. Responders highlighted public and patient partners' lack of understanding of numerical aspects or trial processes as a barrier and suggested solutions to overcoming it such as the use of visual aids or clear language.

"[What would remind me of doing PPI in numerical aspects of trials is…] A better understanding of what they can bring to other numerical aspects (other than oversight committees)." [Trial Manager , involves]

For peer review only - http://bmjopen.bmj.com/site/about/guidelines.xhtml

"Visual aids (e.g. graphs) are useful. Someone on hand to answer questions and to help interpret the information provided." [Trial Manager, involves]

Skills (ability or proficiency to involve patients and the public in numerical aspects of trials)

Respondents who involved public and patient partners in numerical aspects scored higher in the skills domain (4 vs 3 out 7 (feeling able to involve public and patient partners in numerical aspects) for those that involve and those that don't, respectively). However, the answers to open questions relevant to the skills domain focused on patients and the publics' perceived lack of appropriate skills as a cause of concern and barrier to involve them in numerical aspects of trials.

"***[What makes involving public and patient partners in numerical aspects hard is public and patient partners…]*** *Sufficient numeracy skills to understand simple questions around proportions etc*" [Statistician*, does not involve*]

Half of the respondents believed public and patient partners need to have a set of experiences or skills to be involved in numerical aspects of trials (44% (n=52)) of trialists that involve public and patient partners in numerical aspects believed this versus 58% of those that do not (n=41)). When asked to specify what skills public and patient partners would need, some respondents focused on general skills like lived experience of disease or communication. Other respondents thought public and patient partners had to have technical skills in order to be involved, such as numeracy or mathematical skills, statistical understanding, data interpretation and understanding of graphs, knowledge of trial methodology, trial processes and research in general.

Social or professional role (perception of own role and responsibilities in relation to involving patients and the public in numerical aspects of trials)

Forty percent of respondents that involved public and patient partners in numerical aspects considered it an expected role within their jobs (n=46) vs 4% of those that did not involve public and patient partners (n=3). This was supported by qualitative data that showed that some trialists felt like other colleagues, including the Chief Investigator or statistician, would have to drive this process.

"[A barrier to involving public and patient partners in numerical aspects of trials is…] It is not spoken about much and does not always relate directly to job roles." [Statistician, not sure about involvement]

Beliefs about capability (self-belief or confidence in the ability to involve patients and the public in numerical aspects of trials)

Beliefs about trialists' own capability did not seem to drive behaviour with, for example, a similar percentage of responders finding the task difficult in those that did (n=52, 45%) and those that did not (n=34, 48%) involve public and patient partners. However, responses to open questions highlighted trialists' beliefs about public and patient partners capability as a barrier suggesting public and patient partners feel intimidated by numbers, do not grasp them or do not feel confident enough to comment.

"[Two public and patient partners are involved in sample size considerations and recruitment figures…] Both are already confident with numbers/figures so only needed some support with specifics of presentation/meaning." [Trial Manager, involves]

Beliefs about consequences (beliefs about outcomes of involving patients and the public in numerical aspects of trials)

For peer review only - http://bmjopen.bmj.com/site/about/guidelines.xhtml

More than half of respondents that involved public and patient partners in numerical aspects believe there is a good balance between challenges and benefits of doing so (n=68), whereas only 30% shared that belief in those that did not involve (n=22). Responses from open questions showed suggestions of positive consequences: focused on the project (such as improving the interpretation of results, data collection tools and processes, dissemination, trial recruitment, understanding of missing data, interpretation of clinical significance) or the public and patient partners (leads to better understanding of the data and trial processes, more motivated public and patient partners and better team communication). Some responders suggested negative or no consequences such as the impact on public and patient partners being unclear or stressful; a disbelief that it can result in improved trial quality; and extra work and responsibilities for the researcher.

"[Involving public and patient partners in numerical aspects of trials can have…] Multiple impacts, most importantly allowing trial teams to best understand how the key messages of a study can be translated directly to those patients living with condition under study." [Statistician, involves]

Reinforcement (whether there is external stimulus to involve patients and the public in numerical aspects of trials)

A third of responders believed there was recognition from public and patient partners when involving them in numerical aspects of trials if they did so already (n=35) vs 17% for respondents that did not (n=12). Data from open questions supported these observations with trialists mentioning a lack of tangible rewards or encouragement as barriers to doing involving public and patient partners in numerical aspects.

Environmental context and resources (factors related to the work setting that influence involving patients and the public in numerical aspects of trials)

Less than half of trialists felt like they had the resources to support public and patient involvement in numerical aspects if they were doing it (n=55, 47%) and only 23% (n=16) if they were not. This was illustrated in several replies to the open questions with respondents suggesting more resources (financial, human) could facilitate involvement, as well as support identifying interested public and patient partners.

Social influences (perception of how others see one's role and how it impacts upon the ability to involve patients and the public in numerical aspects of research)

Ninety four per cent of those that involve public and patient partners in numerical aspects believe there is some type of encouragement from others to do so (n=7) versus 80% of those that do not involve public and patient partners (n=14). Forty percent of those that involve public and patient partners believe that no one acted as a barrier to do the target behaviour (n=47) vs 18% of those that do not involve public and patient partners (n=13). Qualitative data showed support from colleagues as a team effort, encouragement from management or from public and patient partners were important factors in involving them in numerical aspects of trials.

"Operationally, I don't think PPI members really add a great deal. I also don't think they tend to be interested in those [numerical] aspects. This may be because of the public perception of data and statistics as being hard/obtuse/esoteric." [Trial Manager & statistician, involves]

For peer review only - http://bmjopen.bmj.com/site/about/guidelines.xhtml

Behavioural regulation (perception of guidance or tools to manage or change patient and public involvement in numerical aspects of trials)

About a third of those that involve public and patient partners in numerical aspects state they have a clear plan about which aspects to select, how and when compared with around 7% of those that do not involve public and patient partners. Replies to open questions showed trialists suggest the need for clear guidance on how to conduct PPI in numerical aspects could help facilitate the behaviour, as well as having it as a requirement on ethics committees or standard operating procedures.

"… REC [Research ethics committee] could remind when providing the final report to consider this. The Funder often requires a report so they too could advise to do this. Being reminded by bodies that you have a regulatory or contractual obligation would help. May be journals could advise that this is done in their Instructions to Authors. The RDS also has PPI representatives on their teams and they could act in a capacity to remind people to do this. Internally in my unit, we could do with a guidance document about this." [Trial Manager, involves]

Trial communication culture

Several comments from trialists talked to a broader issue with trial communication culture that highlighted the challenges with communicating clearly about numerical aspects with the entire trial team and ensuring everyone, including patient partners, fully understand them.

"Numbers are presented to the PPI partners all the time in every trial ... So they are involved, but it is by no means actually clear if they or indeed anybody else on the trial team actually understands the figures and the way they are presented. Are they actually any different from anybody else on the trial team? Would improve transparency of decision making which I think would be an improvement even if the actual decisions within the study do not qualitatively change" [Chief Investigator, involves]

Discussion

We have conducted a large UK-based survey to identify current practice, barriers and facilitators of involving public and patient partners in numerical aspects of trials using a behavioural perspective. Because numerical aspects are central to the design, conduct, analysis and dissemination of trials our study explicitly asks about involvement in such aspects rather than more broadly. Our sample represented a wide range of ages, professions and geographic regions in the UK. We used a theory-informed approach to design and analyse our findings to identify barriers and facilitators to involvement of public and patient partners in numerical aspects of trials. We found, in general, trialists believe involving public and patient partners in numerical aspects of trials is a good thing to do but reported several barriers to doing it.

Around 60% of respondents to the survey reported to involve public and patient partners in numerical aspects of trials: a similar percentage of women (62%) reported involving public and patient partners compared with men (66%); there was a lower percentage of early career trialists (up to 5 years of experience) doing this (49%) compared with more experienced trialists (72 and 63% for trialists with 5-10 years of experience and over 10 years, respectively) This contrasts with the report that female and junior researchers are often tasked with doing PPI in health research [3], although it could be an artifact of responders that are particularly interested in PPI. There is a higher proportion of trialists involving public and patient partners in surgical trials (92%,[4]) or even in all trials, ten or more years ago (75%, [5]) than the proportion we found involving public and patient partners in numerical

For peer review only - http://bmjopen.bmj.com/site/about/guidelines.xhtml

aspects. Most of our respondents involved public and patient partners in numerical aspects at the trial design (68%) or dissemination (63%) stages with a significant percentage of respondents involving public and patient partners in undertaking the trial (44%) and in its analysis (35%). Crocker et al survey of PPI in surgical trials [4] and Raza et al. audit of trials in the Integrated Research Application System (IRAS) [13] showed very similar patterns, although both observed a lower prevalence of involvement in the undertaking of a trial. Since involving patients and public partners in oversight committees is common practice [2] but was not a stand-alone option in our survey, it is possible respondents included it as part of trial conduct.

There is scope to improve trialists' knowledge, environmental context and resources and behavioural regulation regarding involving public and patient partners in numerical aspects of trials. Common reflections from the open questions revealed the need for training, more funding, and resources such as finding public and patient partners that are interested in being involved in numerical aspects of trials; developing guidelines about how and when public and patient partners can be involved in numerical aspects would be a natural next step. Trialists have also reported low recognition from public and patient partners, work peers and their managers. These findings are in line with other studies focused on general PPI in research [3], [14] and with wider PPI training needs requested by researchers working in clinical trials in the UK [15]. However, this need was bigger in trialists that did not involve public and patient partners in numerical aspects of trials.

There was a clear difference in the perceived balance of benefits versus challenges of PPI in numerical aspects of trials between those that reported they did it (over 50% reported it as a positive balance) and those that did not (around 30% said the same). This is expected as professionals enacting a behaviour will tend to perceive more benefits than challenges compared with those that are not doing it. Responders to the survey reported more accessible and implementable findings, the opportunity of questioning researchers and more transparent partnerships between researchers and public and patient partners as benefits of involving public and patient partners in numerical aspects of trials. However, some responders saw this as an extra time-consuming task with no evidence of improving the decision making in trials and/or having an impact of patient partners. It is possible these barriers appear more salient to those not involving patient and public partners in numerical aspects and the reasons for that can be varied including their own workplace culture. This is reflected in numerous other studies about researchers' motivations to involve patients and the public as partners in research and the ongoing debate about measurable impact [3], [16].

It is unclear who should be responsible for the involvement in numerical aspects of trials, given chief investigators, statisticians and health economists traditionally have the role of analysing or sharing trial data and results but trial managers are more often in charge of delivering PPI. This perception was echoed in the qualitative data where junior researchers, trial managers and qualitative researchers suggested it was not their responsibility or it was difficult for them to involve public and patient partners in numerical aspects of trials. This was clearly an important factor in the responders' behaviour, since only three respondents (4%) considered involving public and patient partners in numerical aspects of trials as part of their role from trialists that were not currently performing the behaviour (vs 40% of those that did it).

There is uncertainty about public and patient partners' motivation to get involved in numerical aspects of research. Around half of the respondents that involved public and patient partners in numerical aspects and 20% of trialists that don't perceived public and patient partners has being motivated to

For peer review only - http://bmjopen.bmj.com/site/about/guidelines.xhtml

get involved. The thematic analysis identified trialists perceived patient partners lack of interest or finding numbers “boring” as a barrier to involving them in numerical aspects of trials. Our survey included only four public and patient partners respondents and, therefore, it cannot draw conclusions from their perspective but there is scope for more research understanding public and patient partners’ expectations when it comes to involvement in numerical aspects of trials. However, it seems unlikely trialists could confidently speak on behalf of their public and patient partners in terms of motivation to get involved in specific trial aspects. This is because a key challenge raised by several open question comments is the lack of definition of expectations about public and patient partners from the start. Defining expectations is not common practice, even though it is considered useful by all stakeholders [4], [15].

Tokenism and the idea that public and patient partners can only contribute to a limited number of tasks related to increasing readability may be a significant barrier to involving public and patient partners in numerical aspects of trials. A lot of respondents perceived numeracy and statistical knowledge as essential for public and patient partners to get involved in numerical aspects of RCTs and, in some cases, advanced knowledge about sample size calculations and analysis. This contrasts with the fact that public and patient partners often get involved in qualitative research and reinforces perceptions of disciplinary hierarchies [3], but could also be related to the fact that PPI and qualitative research have many common features and therefore seems a natural crossover [17]. In fact, Boylan et al. reported an assumption from UK researchers that the involvement of patients in qualitative interviewing and analysis is uncontroversial; some of our respondents have a clearly different view when it comes to public and patient partners involvement in quantitative analysis. From the public and patient partners perspective, a respondent pointed out that they often felt underestimated and another that tokenism is the main barrier to involving public and patient partners in numerical aspects. Wider literature on the topic shows there are appropriate methods to involve non-experts in discussions about statistical models and numbers [6], although this is a controversial topic [7]. Either way, communication, in particular about numerical aspects of trials, needs to improve to enable meaningful PPI in trials and this has been known for almost a decade [5] and it is a key priority for future PPI research according to multiple stakeholders [18]. A way forward could, for example, follow models to achieve better communication related to patient data, such as the Understanding Patient Data project (https://understandingpatientdata.org.uk/).

Limitations

Like with most behaviours, involving public and patient partners in numerical aspects of trials is a behaviour in a spectrum. Respondents to our survey interpreted it in a variety of ways – ranging from presenting numbers in reports to public and patient partners at trial steering meetings to involving them in doing the statistical analysis. Despite the iterative development of a definition endorsed by multiple trial stakeholders and its presentation at the beginning of the survey, it is likely that the barriers and facilitators presented here regard a range of behaviours in that spectrum. To develop the survey, our patient and public involvement was limited to piloting. Since the survey aimed to understand practices, barriers and facilitators for professionals, we considered this to be sufficient within the context of a Masters’ student project, but patient and public partners’ perspective could have enriched the study questions and survey design and potentially increased the number of patient and public partners responding. We did not include a stand-alone option of PPI in numerical aspects of data and trial steering committees; however, we do not consider this to be essential information to

For peer review only - http://bmjopen.bmj.com/site/about/guidelines.xhtml

our purpose (a broad overview of PPI in numerical aspects of trials) and, nevertheless, respondents added this information in open questions. Qualitative analysis was based on open questions in a survey which are limited in their scope when compared to conducting interviews and may lead to misinterpretation of answers [19]. We tried to minimise this by double assessing a subsample of qualitative data. Our sample was diverse and coming from multiple locations and professions across the country. Respondents that answered demographic questions but did not proceed to answer questions specifically about PPI in numerical aspects of trials were mostly similar to those that did; however, we are likely to have respondents that have stronger views of PPI and PPI in numerical aspects of trial since they opted to answer our survey.

Conclusion

We found a generally positive attitude towards involving public and patient partners in numerical aspects of trials with 60% of the respondents reporting attempting to do so in a variety of ways. However, there is a need to understand public and patient partners expectations when getting involved in a trial and whether they would have an interest in being involved in numerical aspects. To achieve that, public and patient partners first need to be aware of the different stages of trials and the opportunities to get involved. Numerical aspects are a key element in trials and, therefore, should at least be part of an initial discussion. Our survey has also highlighted that communication of numerical and methodological aspects of trials can be challenging, not just for public and patient partners but for the whole trial team. Improving it should be a matter of providing training on either communication skills, technical aspects or both and it should be available for the whole team, including but not limited to public and patient partners. A training set co-designed by relevant stakeholders and available to all clinical trials units in the UK, including successful case studies of involving public and patient partners in numerical aspects of trials, is an essential step forward, as well as guidance on what are the most relevant numerical aspects of trials to involve public and patient partners in and how to do that in a meaningful way.

Acknowledgments: We would like to thank staff and student members at the University of Aberdeen who helped pilot test the survey and, in particular, to Dr Eilidh Duncan for her expertise and support in the development of the questions underpinned by the theoretical domains framework. We would like to acknowledge all trialists that took the time to reply to this survey and everyone that helped disseminate it.

Author Contributors: CP wrote the first draft of the protocol. BG and KG contributed to the design of the project and development of the protocol. BG and CP conducted the quantitative analysis; BG conducted the qualitative analysis; KG checked a sample of BG's analysis as a quality check. BG wrote the first of the manuscript. All authors approved the final version of the manuscript.

Funding: The first author was supported to develop this research by the Wellcome Trust – Institutional Strategic Support Fund at the University of Aberdeen.

Conflicts of interest: The authors have no conflicts of interest to declare.

Patient consent form: Not applicable

Data availability statement: Data are available upon reasonable request.

Word count: 5379

For peer review only - http://bmjopen.bmj.com/site/about/guidelines.xhtml

Exclusive licence

I, the Submitting Author has the right to grant and does grant on behalf of all authors of the Work (as defined in the below author licence), an exclusive licence and/or a non-exclusive licence for contributions from authors who are: i) UK Crown employees; ii) where BMJ has agreed a CC-BY licence shall apply, and/or iii) in accordance with the terms applicable for US Federal Government officers or employees acting as part of their official duties; on a worldwide, perpetual, irrevocable, royalty-free basis to BMJ Publishing Group Ltd ("BMJ") its licensees and where the relevant Journal is co-owned by BMJ to the co-owners of the Journal, to publish the Work in BMJ Open and any other BMJ products and to exploit all rights, as set out in our licence.

The Submitting Author accepts and understands that any supply made under these terms is made by BMJ to the Submitting Author unless you are acting as an employee on behalf of your employer or a postgraduate student of an affiliated institution which is paying any applicable article publishing charge ("APC") for Open Access articles. Where the Submitting Author wishes to make the Work available on an Open Access basis (and intends to pay the relevant APC), the terms of reuse of such Open Access shall be governed by a Creative Commons licence – details of these licences and which Creative Commons licence will apply to this Work are set out in our licence referred to above.

References

[1] National Institute for Health Research, "Patient and Public Involvement in Health and Social Care Research: A handbook for researchers," pp. 1–40, 2013.

[2] A. Price *et al.*, "Patient and public involvement in the design of clinical trials: An overview of systematic reviews," *J. Eval. Clin. Pract.*, vol. 24, no. 1, pp. 240–253, 2018, doi: 10.1111/jep.12805.

[3] A. M. Boylan, L. Locock, R. Thomson, and S. Staniszewska, "'About sixty per cent I want to do it': Health researchers' attitudes to, and experiences of, patient and public involvement (PPI)—A qualitative interview study," *Heal. Expect.*, vol. 22, no. 4, pp. 721–730, 2019, doi: 10.1111/hex.12883.

[4] J. C. Crocker *et al.*, "Patient and public involvement (PPI) in UK surgical trials: A survey and focus groups with stakeholders to identify practices, views, and experiences," *Trials*, vol. 20, no. 1, pp. 1–15, 2019, doi: 10.1186/s13063-019-3183-0.

[5] C. Gamble *et al.*, "An evidence base to optimise methods for involving patient and public contributors in clinical trials: a mixed-methods study," *Heal. Serv. Deliv. Res.*, vol. 3, no. 39, 2015, doi: 10.3310/hsdr03390.

[6] A. Hannigan, "Public and patient involvement in quantitative health research : A statistical perspective," *Heal. Expect.*, no. May, pp. 1–5, 2018, doi: 10.1111/hex.12800.

[7] G. A. K. van Voorn *et al.*, "The Missing Stakeholder Group: Why Patients Should be Involved in Health Economic Modelling," *Appl. Health Econ. Health Policy*, vol. 14, no. 2, pp. 129–133, 2016, doi: 10.1007/s40258-015-0200-7.

[8] J. Cane, D. O'Connor, and S. Michie, "Validation of the theoretical framework," *Implement. Sci.*, vol. 7, p. 37, 2012.

[9] J. Presseau *et al.*, "Barriers and facilitators to healthcare professional behaviour change in

For peer review only - http://bmjopen.bmj.com/site/about/guidelines.xhtml

clinical trials using the Theoretical Domains Framework: A case study of a trial of individualized temperature-reduced haemodialysis," *Trials*, vol. 18, no. 1, pp. 1–16, 2017, doi: 10.1186/s13063-017-1965-9.

[10] W. Skoien, K. Page, W. Parsonage, S. Ashover, T. Milburn, and L. Cullen, "Use of the Theoretical Domains Framework to evaluate factors driving successful implementation of the Accelerated Chest pain Risk Evaluation (ACRE) project," *Implement. Sci.*, vol. 11, no. 1, pp. 1–11, 2016, doi: 10.1186/s13012-016-0500-9.

[11] J. M. Huijg *et al.*, "Measuring determinants of implementation behavior: Psychometric properties of a questionnaire based on the theoretical domains framework," *Implement. Sci.*, vol. 9, no. 1, pp. 1–15, 2014, doi: 10.1186/1748-5908-9-33.

[12] R. Newlands *et al.*, "Barriers and facilitators of evidence-based management of patients with bacterial infections among general dental practitioners: A theory-informed interview study," *Implement. Sci.*, vol. 11, no. 1, pp. 1–12, 2016, doi: 10.1186/s13012-016-0372-z.

[13] M. Z. Raza, H. Bruhn, and K. Gillies, "Dissemination of trial results to participants in phase III pragmatic clinical trials: An audit of trial investigators intentions," *BMJ Open*, vol. 10, no. 1, pp. 1–8, 2020, doi: 10.1136/bmjopen-2019-035730.

[14] K. McKenzie, A., Bulsara, C., Haines, H., Hanley, B., Alpers, *Barriers to community involvement in health and medical research*. 2016.

[15] L. Dudley *et al.*, "A little more conversation please? Qualitative study of researchers' and patients' interview accounts of training for patient and public involvement in clinical trials," *Trials*, vol. 16, no. 1, pp. 1–15, 2015, doi: 10.1186/s13063-015-0667-4.

[16] I. Schilling *et al.*, "Patients' and researchers' experiences with a patient board for a clinical trial on urinary tract infections," *Res. Involv. Engagem.*, vol. 5, no. 1, pp. 1–14, 2019, doi: 10.1186/s40900-019-0172-0.

[17] L. Locock and A. Boaz, "Drawing straight lines along blurred boundaries: Qualitative research, patient and public involvement in medical research, co-production and co-design," *Evid. Policy*, vol. 15, no. 3, pp. 409–421, 2019, doi: 10.1332/174426419X15552999451313.

[18] A. Kearney *et al.*, "Priorities for methodological research on patient and public involvement in clinical trials: A modified Delphi process," *Heal. Expect.*, vol. 20, no. 6, pp. 1401–1410, 2017, doi: 10.1111/hex.12583.

[19] V. Braun and V. Clarke, *Succesful qualitative research*. SAGE, 2013.

For peer review only

For peer review only - http://bmjopen.bmj.com/site/about/guidelines.xhtml

Table 1 – Descriptive statistics by trialists current practice (involving PPP in numerical aspects of trials or not / not sure) and in total – values are n(%)

	Trialists that involve PPP in numerical aspects (N=116)	Trialists that do not involve PPP in numerical aspects or don't know (N=71)	Total
**Age**			
18-24 years old	1 (0.9)	1 (1.4)	2 (1.1)
25-34 years old	32 (27.6)	30 (42.3)	62 (33.2)
35-44 years old	38 (32.8)	12 (16.9)	50 (26.7)
45-54 years old	25 (21.6)	16 (22.5)	41 (21.9)
55-64 years old	14 (12.1)	8 (11.3)	22 (11.8)
65+ years old	5 (4.3)	2 (2.8)	7 (3.7)
Rather not say	1 (0.9)	2 (2.8)	3 (1.6)
**Gender**			
Female	80 (69.0)	50 (70.4)	130 (69.5)
Male	33 (28.4)	17 (23.9)	50 (26.7)
Other		2 (2.8)	2 (1.1)
Prefer not to say	3 (2.6)	2 (2.8)	5 (2.7)
**Work location**			
England	9 (7.8)	8 (11.3)	17 (9.1)
North East England	4 (3.4)	3 (4.2)	7 (3.7)
North West England	7 (6.0)	7 (9.9)	14 (7.5)
Yorkshire and the Humber	8 (6.9)	5 (7.0)	13 (7.0)
West Midlands	10 (8.6)	2 (2.8)	12 (6.4)
East Midlands	3 (2.6)	4 (5.6)	7 (3.7)
South West England	9 (7.8)	2 (2.8)	11 (5.9)
South East England	13 (11.2)	6 (8.5)	19 (10.2)
London	19 (16.4)	14 (19.7)	33 (17.6)
East England	3 (2.6)	1 (1.4)	4 (2.1)
Northern Ireland	3 (2.6)	3 (4.2)	6 (3.2)
Scotland	18 (15.5)	12 (16.9)	30 (16.0)
Wales	10 (8.6)	4 (5.6)	14 (7.5)
**Which aspects of the trial process are you directly involved in?**			
Trial design	51 (44.0)	37 (52.1)	88 (47.1)
Undertaking the trial	48 (41.4)	37 (52.1)	85 (45.5)
Dissemination	55 (47.4)	34 (47.9)	89 (47.6)
Analysis	36 (31.0)	23 (32.4)	59 (31.6)
Overseeing committee	1 (0.9)	4 (5.6)	5 (2.7)
All stages	56 (48.3)	19 (26.8)	75 (40.1)
**Main role in trial**			
Trial manager	81 (69.8)	44 (62.0)	125 (66.8)
Chief investigator	2 (1.7)	1 (1.4)	3 (1.6)
Statistician	16 (13.8)	13 (18.3)	29 (15.5)
Health economist	2 (1.7)	1 (1.4)	3 (1.6)

For peer review only - http://bmjopen.bmj.com/site/about/guidelines.xhtml

Qualitative researcher	6 (5.2)	2 (2.8)	8 (4.3)
Patient Partner	4 (3.4)	4 (5.6)	8 (4.3)
Co-investigator	9 (7.8)	7 (9.9)	16 (8.6)
Researcher	6 (5.2)	1 (1.4)	7 (3.7)
Systematic reviewer		1 (1.4)	1 (0.5)
PPI lead	1 (0.9)	2 (2.8)	3 (1.6)
Not reported	1 (0.9)		1 (0.5)
**How long have you been working in trials?**			
Less than 5 years	28 (24.1)	29 (40.8)	57 (30.5)
Between 5-10 years	44 (37.9)	17 (23.9)	61 (32.6)
More than 10 years	44 (37.9)	25 (35.2)	69 (36.9)
**When do you involve PPP in numerical aspects of trials?**			
Never	2 (1.7)		2 (1.1)
Rarely	10 (8.6)		10 (5.3)
Regularly	72 (62.1)		72 (38.5)
Every day	32 (27.6)		32 (17.1)
**What stage of a trial do you involve PPP in the numerical aspects?**			
Trial design	86 (74.1)		86 (46.0)
Undertaking the trial	58 (50.0)		58 (31.0)
Dissemination	84 (72.4)		84 (44.9)
Undertaking the trial	58 (50.0)		58 (31.0)
Overseeing committee	8 (6.9)		8 (4.3)
No information available	2 (0.9)		4 (1.6)

For peer review only

For peer review only - http://bmjopen.bmj.com/site/about/guidelines.xhtml

Table 2 – Responses to selected Theoretical Domains Framework (TDF) questions by whether respondent said they involved patient and public partners (PPP) in numerical aspects of trials – median (percentile 25 – percentile 75), count for scale variables or n (%) out of N for binary variables

	Respondents involve PPP (N=116)	Respondents do not involve PPP or are not sure (N=71)
**TDF domain:** Knowledge		
How familiar are you with involving PPP in numerical aspects of trials?	4 (3.0-5.0),116	2 (1.0-3.0),71
**TDF domain:** Skills		
Grade your own ability to involve PPP in numerical aspects of trials	4 (3.0-5.0),116	2 (1.0-4.0),71
**PPP skills**		
**Do you think the PPP need to have particular experience or skills to be involved in numerical aspects of trials?**		
Yes	52 (44.8)	41 (57.7)
No	64 (55.2)	30 (42.3)
**TDF domain:** Social / professional role		
Is involving PPP in numerical aspects of trials an expected role within your job?	46 (39.7)	3 (4.2)
**TDF domain:** Beliefs about capability		
Do you feel confident in your ability to involve PPP in numerical aspects of trials?	4 (3.0-5.0),111	3 (2.0-4.0),62
Do you think that involving PPP in numerical aspects of trials is hard to deliver?	52 (44.8)	34 (47.9)
**TDF domain:** Beliefs about consequences		
There is a good balance between the challenges of involving PPP in numerical aspects of trials and the potential benefits	68 (58.6)	22 (31.0)
**TDF domain:** Reinforcement		
You get recognition from PPP, when you involve them in numerical aspects of trials	35 (30.2)	12 (16.9)
You get recognition from work peers	21 (18.1)	5 (7.0)
You get recognition from your manager	23 (19.8)	6 (8.5)
**TDF domain:** Environmental context and resources		
Have the resources needed to involve PPP in numerical aspects of trials	55 (47.4)	16 (22.5)
Employer provides support to involve PPP in numerical aspects of trials	47 (40.5)	13 (18.3)
Employer provides training to involve PPP in numerical aspects of trials	31 (26.7)	7 (9.9)
Involving PPP in numerical aspects of trials is compatible with daily practice	63 (54.3)	17 (23.9)
PPP are motivated to get involved in numerical aspects of trials	59 (50.9)	14 (19.7)
**TDF domain:** Social influences		
Most people who are important think I should involve PPP in numerical aspects of trials	5 (4.0-6.0),94	3 (1.0-4.0),50

For peer review only

For peer review only - http://bmjopen.bmj.com/site/about/guidelines.xhtml

**Who encourages you to involve PPP in numerical aspects?**		
No encouragement	7 (6.0)	14 (19.7)
**Who is a barrier for you to involve PPP in numerical aspects?**		
No barrier	47 (40.5)	13 (18.3)
**TDF domain:** Behavioural regulation		
**Do you have a clear plan on…**		
Which numerical aspects of trials you should involve PPP with?	33 (28.4)	5 (7.0)
How you will involve PPP in numerical aspects of trials?	36 (31.0)	6 (8.5)
How often will you involve PPP in numerical aspects of trials?	37 (31.9)	4 (5.6)

For peer review only

Table 3 – Open question responses coded by domain and grouped according to specific beliefs

Domains	Specific belief	Illustrating quotes
Knowledge	Lack of clarity about what the task entails	*“[What would help me involve PPP in numerical aspects of trials is…] Understanding what involving patients and public in numerical aspects of trials actually means, how it can be done and why it's important” [TM, NI]*<br><br>*“I think the main issue here is that there are very simple parts of numerical aspects of trials which we would always, and easily, involve PPI in: outcome measure acceptability and monitoring trial progress. I don't know what other aspects might be, so I am imagining a training or knowledge failure on my part.” [TM, involves]*
	Training	*“[What would help me involve PPP in numerical aspects is…] training courses/workshops for members of the public on the numerical aspects of trials and also researchers on how to involve them” [HE, NI]*<br><br>*“This is something that has never really been discussed at work. Nor have I seen any courses about doing this. More guidance is available about writing simply for patients but not specifically about numerical aspects. Some training would be welcome.” [TM, involves]*
	Knowledge of patient and public partners	*“[What makes involving PPP in numerical aspects hard is…] Being confident patients have sufficient understanding to be able to contribute. Not sure what could make it easier, just need to be aware this may be a potential issue during initial discussions with focus group members for example.” [TM, involves]*<br><br>*“[What makes involving PPP in numerical aspects hard is…] a general lack of awareness of how trials (and research) work, which makes the initial engagement harder. It's perfectly possible to overcome this though.” [Qualitative researcher, involves]*

For peer review only - http://bmjopen.bmj.com/site/about/guidelines.xhtml

Skills	Skills of patient and public partners	*"... I would suggest that if you want to involve PPI in this [numerical] aspect then you would have to purposively approach PPI with a certain skill set or advertise for such PPI involvement" [Co-investigator, NI]*<br><br>*"It generally quite boring for the general public and they do need a basic level of numeracy and appreciation of research methods" [Statistician, involves]*
Social / professional role	Professional role	*"The grant applicants and senior team often take these decisions during trial design. I never had any impact on these decisions previously." [Qualitative researcher, involves]*<br><br>*"Numerical roles already 'assigned' to statisticians and clinicians, so as Trial Manager, it can be difficult to enforce PPI involvement" [TM, involves]*
Beliefs about capability	Capabilities of patient and public partners	*"Often numerical aspects are dictated by statistical programs and I think there is a general feeling that patient or public partners cannot grasp them." [TM, NI]*<br><br>*"[A barrier to involve PPP in numerical aspects of trials is…] The PP themselves not being confident enough to comment" [Co-investigator, involves]*
Beliefs about consequences	Impact on trial quality	*"Patients can help you work out how to disseminate the results e.g. which graphs are easiest, which format of presenting an estimate (risk, number of days etc)" [Statistician, involves]*<br><br>*"Discussing numerical information to patients helps to distill complex findings into something more simple and acceptable. This helps the researcher to clearer think through what are the results, helps explain information clearly that improves the quality of the trial and will ultimately all benefit the patient." [TM, involves]*

For peer review only - http://bmjopen.bmj.com/site/about/guidelines.xhtml

	Positive impact on patient and public partners	*"The PPP can … increase their knowledge and understanding of their condition, new potential treatments, and the process involved in trials which evaluate treatments. This could reduce the information asymmetry between the patient and clinician in the future" [Health Economist, involves]*<br><br>*"Patients can understand the difficulties in running clinical research and trials. Showing recruitment figures and follow-up data is also beneficial as patients can see the results accumulating and the benefits of taking part in trials." [TM, involves]*
	Negative impact on patient and public partners	*"Makes participation stressful, unless the PP has pre-existing skills in stats or maths." [Co-investigator, involves]*
	Extra work / responsibilities for the researcher	*"It is more work for the researcher to involve patients/partners" [Statistician, NI]*
	Uncertainty about impact on trial quality	*"I think it could improve transparency of decision making through a trial process. I am less sure that will actually translate into "better" decisions" [CI, involves]*<br><br>*"I have answered no impact because I think it is unclear whether quality or the patient partner will get a positive impact. That is a question that needs investigation." [CI, involves]*
Reinforcement	Lack of incentives	*"Having some tangible reward for your investment! It never does anything for your career, while other priorities do." [Statistician, involves]*<br><br>*"It's not really rewarded or recognised because it is an expected part of study design. All funders we work with consider PPI involvement with trials in general essential." [TM & statistician, involves]*
Environmental context and resources	Funding	*"[What would help me involve PPP in numerical aspects is…] More funding for a PPI expert at our CTU. I am only funded 10% to be the coordinator for all PPI and to do a good job, I'd like to be able to spend more time on my PPI role" [TM, involves]*
	Time	*"I will need training to effectively include the public and the public will need training to effectively contribute to the research. This will take more time*

For peer review only - http://bmjopen.bmj.com/site/about/guidelines.xhtml

		and resources that aren't currently provided but if they were this would make it much easier" [HE, NI]
	Staff	*"[What would help me involve PPP in numerical aspects is…] More money, more trained help. The usual problems." [TM, involves]*<br><br>*"[What would help me involve PPP in numerical aspects is…]* Having a special person dedicated to running PPI work across trials." *[TM, involves]*
	Difficulty accessing willing volunteers	*"It's difficult to find public partners for any research, but when it's something that's specifically numerical I think that's an even bigger challenge!" [Qualitative researcher & TM, involves]*<br><br>*"[What would help me involve PPP in numerical aspects is…] To know how or where to find participants that would be interested in participate." [TM, NI]*
Social influences	Supportive team environment	*"[What would help me involve PPP in numerical aspects is…] More of a group/team effort so that it is not just left to the trial manager to present to PPI members but the trial team as a whole takes on this responsibility." [TM, involves]*<br><br>*"I think the enthusiasm of the chief investigator to include PPI members is important. If the investigator wants to involve PPI then it will happen but if the investigator is not bothered then it proves much more difficult to include someone." [TM, involves]*
	Patient and public partners' interest	*"I would be happy to share details of the numerical analysis, but have never been asked to do so - they seem satisfied with the summary explanations provided." [TM, NI]*<br><br>*"PPs generally shrink back even when asked to comment on numerical aspects of a trial" [Co-investigator, involves]*

For peer review only

For peer review only - http://bmjopen.bmj.com/site/about/guidelines.xhtml

Behavioural regulation	Guidance	*"… If there was clear guidance and tools on how to do it and those tools could be easily integrated into management structures for running large scale studies then I would use them" [CI, involves]*<br><br>*"[What would help me involve PPP in numerical aspects is…] Guidelines about PPI expectations for review of numerical aspects" [TM, unsure about involvement]*
	Standard Operating Procedures	*"[What would help me involve PPP in numerical aspects is…] have this implemented into SOPs [Standard Operating Procedures]" [TM, NI]*
Trial communication culture	Lack of clarity in communication about numerical and statistical aspects of trials	*"language and jargon used around sample size calculations etc in design discussions. Stats/methodologists need to be open and able to discuss complex issues openly and simply" [TM, involves]*<br><br>*"As a patient partner I feel very privileged … But as a retired maths teacher, I am very aware of the anxiety that numbers cause even otherwise very clever people - these anxieties will only be mitigated by more involvement at all levels, including the public." [PPP, involves]*<br><br>*"If everyone in the research environment explained what the numbers mean clearly to all members of the team - that would make it easier to describe and facilitate involvement from patient or the public" [TM, NI]*

CI – Chief Investigator; HE – Health Economist; TM – Trial manager; NI – doesn't involve PPP in numerical aspects

Figure legend

Figure 1 – Flow of participants (* we present demographic information by respondent status in Supplementary Table 1). TDF – Theoretical Domains Framework

For peer review only - http://bmjopen.bmj.com/site/about/guidelines.xhtml

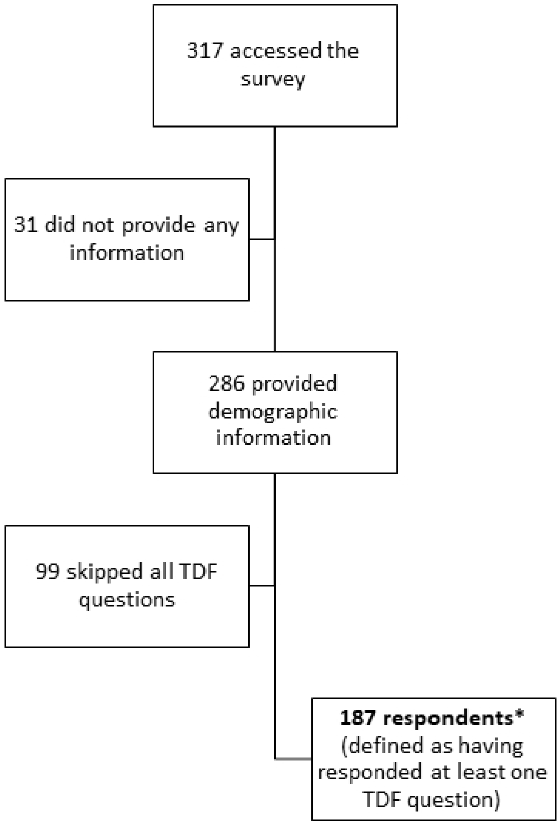

Figure 1 – Flow of participants (* we present demographic information by respondent status in Supplementary Table 1). TDF – Theoretical Domains Framework

Supplementary Table 1 – Demographic characteristics by respondent status (respondents were defined as participants that replied to demographic characteristics and all theoretical domains framework questions; non-respondents were those that replied to demographic questions only)

	Respondents (N=187)	Non-respondents (N=99)
**Age**		
18-24 years old	2 (1.1)	3 (3.0)
25-34 years old	62 (33.2)	27 (27.3)
35-44 years old	50 (26.7)	35 (35.4)
45-54 years old	41 (21.9)	20 (20.2)
55-64 years old	22 (11.8)	9 (9.1)
65+ years old	7 (3.7)	4 (4.0)
Rather not say	3 (1.6)	1 (1.0)
**Gender**		
Female	130 (69.5)	82 (82.8)
Male	50 (26.7)	17 (17.2)
Other	2 (1.1)	
Prefer not to say	5 (2.7)	
**Work location**		
England	17 (9.1)	10 (10.1)
North East England	7 (3.7)	3 (3.0)
North West England	14 (7.5)	7 (7.1)
Yorkshire and the Humber	13 (7.0)	11 (11.1)
West Midlands	12 (6.4)	1 (1.0)
East Midlands	7 (3.7)	6 (6.1)
South West England	11 (5.9)	6 (6.1)
South East England	19 (10.2)	10 (10.1)
London	33 (17.6)	18 (18.2)
East England	4 (2.1)	3 (3.0)
Northern Ireland	6 (3.2)	5 (5.1)
Scotland	30 (16.0)	16 (16.2)
Wales	14 (7.5)	3 (3.0)
**Which aspects of the trial process are you directly involved in?**		
Trial design	88 (47.1)	51 (51.5)
Undertaking the trial	85 (45.5)	50 (50.5)
Dissemination	89 (47.6)	39 (39.4)
Analysis	59 (31.6)	24 (24.2)
Overseeing committee	5 (2.7)	1 (1.0)
All stages	75 (40.1)	25 (25.3)
None		1 (1.0)
**Main role in trial**		
Trial manager	125 (66.8)	69 (69.7)
Chief investigator	3 (1.6)	
Statistician	29 (15.5)	8 (8.1)
Health economist	3 (1.6)	3 (3.0)
Qualitative researcher	8 (4.3)	
Patient Partner	8 (4.3)	3 (3.0)
Co-investigator	16 (8.6)	10 (10.1)

For peer review only - http://bmjopen.bmj.com/site/about/guidelines.xhtml

Researcher	7 (3.7)	6 (6.1)
Systematic reviewer	1 (0.5)	
Programmer		1 (1.0)
PPI lead	3 (1.6)	4 (4.0)
Don't work in a trial		2 (2.0)
Missing	63 (33.2)	30 (30.3)
**How long have you been working in trials?**		
Less than 5 years	57 (30.5)	35 (35.4)
Between 5-10 years	61 (32.6)	28 (28.3)
More than 10 years	69 (36.9)	36 (36.4)
**When do you involve patient or public partners in numerical aspects of trials?**		
Never	7 (3.7)	7 (7.1)
Rarely	23 (12.3)	18 (18.2)
Regularly	109 (58.3)	57 (57.6)
Every day	48 (25.7)	11 (11.1)
Missing		6 (6.1)
**Do you involve patient or public partners in the numerical aspects of trials?**		
Yes	116 (62.0)	18 (18.2)
No	48 (25.7)	6 (6.1)
Don't know	23 (12.3)	3 (3.0)
Missing		72 (72.7)

For peer review only

Supplementary Table 2 – Responses to the Theoretical Domains Framework questions by whether respondent said they involved patient and public partners (PPP) in numerical aspects of trials – median (percentile 25 – percentile 75), count for scale variables or n (%) out of N for binary variables

	Respondents involve PPP (N=116)	Respondents do not involve PPP or are not sure (N=71)
**TDF domain:** Knowledge		
How familiar are you with involving PPP in numerical aspects of trials?	4 (3.0-5.0),116	2 (1.0-3.0),71
**TDF domain:** Skills		
Grade your own ability to involve PPP in numerical aspects of trials	4 (3.0-5.0),116	2 (1.0-4.0),71
**TDF domain:** Social / professional role		
Is involving PPP in numerical aspects of trials an expected role within your job?	46 (39.7)	3 (4.2)
**TDF domain: Beliefs about capability**		
Do you feel confident in your ability to involve PPP in numerical aspects of trials?	4 (3.0-5.0),111	3 (2.0-4.0),62
Do you think that involving PPP in numerical aspects of trials is hard to deliver?	52 (44.8)	34 (47.9)
**TDF domain:** Optimism		
Involving PPP in numerical aspects of trials is a good thing	6 (5.0-7.0),108	5 (4.0-6.0),61
**Do you think involving patient or partners in numerical aspects of a trial can have a positive impact on the researcher?**		
Yes	96 (82.8)	43 (60.6)
No impact	5 (4.3)	11 (15.5)
Negative impact	1 (0.9)	2 (2.8)
Missing	14 (12.1)	15 (21.1)
**on the quality of the trial?**		
Yes	93 (80.2)	46 (64.8)
No impact	8 (6.9)	9 (12.7)
Negative impact	1 (0.9)	1 (1.4)
Missing	14 (12.1)	15 (21.1)
**on the patient partner?**		
Yes	95 (81.9)	50 (70.4)
No impact	6 (5.2)	4 (5.6)
Negative impact	1 (0.9)	2 (2.8)
Missing	14 (12.1)	15 (21.1)
**TDF domain:** beliefs about consequences		
There is a good balance between the challenges of involving PPP in numerical aspects of trials and the potential benefits	68 (58.6)	22 (31.0)
**TDF domain:** Reinforcement		
You get recognition from PPP, when you involve them in numerical aspects of trials	35 (30.2)	12 (16.9)
You get recognition from work peers	21 (18.1)	5 (7.0)

For peer review only

You get recognition from your manager	23 (19.8)	6 (8.5)
**TDF domain:** Intention		
**Will you involve PPP in numerical aspects of your next project?**		
Yes definitely	41 (35.3)	4 (5.6)
Yes probably	42 (36.2)	16 (22.5)
No definitely	0	1 (1.4)
No probably	4 (3.4)	9 (12.7)
I don't know	12 (10.3)	24 (33.8)
Missing	17 (14.7)	17 (23.9)
**TDF domain:** Goals		
Working on something else on my agenda is a higher priority than involving PPP in the numerical aspects of trials	4 (3.0-5.0),99	5 (4.0-6.0),54
**TDF domain:** Memory, attention, decision process		
Involving PPP in numerical aspects of trials is something I do automatically	4 (3.0-6.0),93	2 (1.0-2.5),52
**TDF domain:** Environmental context and resources		
Have the resources needed to involve PPP in numerical aspects of trials	55 (47.4)	16 (22.5)
Employer provides support to involve PPP in numerical aspects of trials	47 (40.5)	13 (18.3)
Employer provides training to involve PPP in numerical aspects of trials	31 (26.7)	7 (9.9)
Involving PPP in numerical aspects of trials is compatible with daily practice	63 (54.3)	17 (23.9)
PPP are motivated to get involved in numerical aspects of trials	59 (50.9)	14 (19.7)
**TDF domain:** Social influences		
Most people who are important think I should involve PPP in numerical aspects of trials	5 (4.0-6.0),94	3 (1.0-4.0),50
**Who encourages you to involve PPP in numerical aspects?**		
Myself	63 (54.3)	15 (21.1)
Co-workers	45 (38.8)	11 (15.5)
Manager	44 (37.9)	10 (14.1)
Social media	14 (12.1)	6 (8.5)
My personal environment	18 (15.5)	1 (1.4)
Scientific literature	31 (26.7)	15 (21.1)
Public	24 (20.7)	9 (12.7)
Institution	38 (32.8)	10 (14.1)
Regulation	22 (19.0)	15 (21.1)
Mass media	3 (2.6)	3 (4.2)
No encouragement	7 (6.0)	14 (19.7)
**Who is a barrier for you to involve PPP in numerical aspects?**		
Myself	6 (5.2)	7 (9.9)
Co-workers	4 (3.4)	7 (9.9)

For peer review only

Manager	6 (5.2)	10 (14.1)
Scientific literature	2 (1.7)	3 (4.2)
Social media	2 (1.7)	
My personal environment	11 (9.5)	6 (8.5)
Public	5 (4.3)	7 (9.9)
Institution	13 (11.2)	13 (18.3)
Regulation	10 (8.6)	5 (7.0)
Mass media	5 (4.3)	3 (4.2)
No barrier	47 (40.5)	13 (18.3)
**TDF domain:** Emotions		
**Emotions related to involving PPP in numerical aspects**		
Optimism	65 (56.0)	29 (40.8)
Ease	18 (15.5)	5 (7.0)
Calm	14 (12.1)	3 (4.2)
Relaxation	3 (2.6)	3 (4.2)
Appreciation	71 (61.2)	32 (45.1)
Nervousness	37 (31.9)	18 (25.4)
Pessimism	7 (6.0)	12 (16.9)
Depression		2 (2.8)
Agitation	9 (7.8)	11 (15.5)
Happiness	27 (23.3)	8 (11.3)
Sadness	2 (1.7)	2 (2.8)
Anxiety	25 (21.6)	17 (23.9)
**TDF domain:** Behavioural regulation		
**Do you have a clear plan on…**		
Which numerical aspects of trials you should involve PPP with?	33 (28.4)	5 (7.0)
How you will involve PPP in numerical aspects of trials?	36 (31.0)	6 (8.5)
How often will you involve PPP in numerical aspects of trials?	37 (31.9)	4 (5.6)
**TDF domain:** Nature of behaviour		
Involve PPP in numerical aspects of trials without consciously thinking about it	4 (2.0-5.0),90	2 (1.0-3.5),48

For peer review only - http://bmjopen.bmj.com/site/about/guidelines.xhtml

Survey

Demographic questions

**Questions**	**Response options**
**What is your age?**	18-24 years old<br>25-34 years old<br>35-44 years old<br>45-54 years old<br>55-64 years old<br>65-74 years old<br>75 years or older
**Gender: What is your gender?**	Female<br>Male<br>Other<br>Rather not say
**Geographic: Where is your workplace is based?**	England<br>North East England<br>North West England<br>Yorkshire and the Humber<br>West Midlands<br>East Midlands<br>South West England<br>South East England<br>London<br>East England<br>Northern Ireland<br>Scotland<br>Wales
**Job: What is your main role in the trial?**	Trial Manager
	Chief Investigator
	Co-Investigator
	Statistician
	Data manager

For peer review only - http://bmjopen.bmj.com/site/about/guidelines.xhtml

	Patient partner
	Qualitative researcher
	Health Economist
	Don’t work in a trial
	Other
**Job: How long have you worked in clinical trials?**	Less than 5 years<br>Between 5-10 years<br>More than 10 years
**Job: Which aspects of the trials process are you directly involved in?**	Trial design
	Undertaking the trial (e.g. identifying or recruiting participants, collecting data)
	Analysis and/or interpretation of results
	Dissemination of findings
	None of the above
	All of the above
	Other

For peer review only

For peer review only - http://bmjopen.bmj.com/site/about/guidelines.xhtml

Current behavior / beliefs

Questions	Response options
How often are you involved in the numerical aspects of a trial, according to the definition above?	1: never<br>2: rarely<br>3: regularly<br>2: every day<br>0: other
Do you involve patient or public partners in the numerical aspects of trials?	1: yes<br>2: no<br>3: don't know
Can you provide the reasons for why you do, or do not, involve patient or public partners in the numerical aspects of trials?	Open question
What stage of a trial do you involve public or patient partners in the numerical aspects of a trial?	Research design
	Undertaking the research (e.g. Identifying or recruiting participants, collecting data)
	Analysis and/or interpretation of results
	Dissemination of findings
	None of the above

For peer review only

For peer review only - http://bmjopen.bmj.com/site/about/guidelines.xhtml

	Other (please specify)
When you involve patient or public partners in the numerical aspects of a trial, where on the scale do you picture yourself?	0-100 (informing – co-producing)
When involving patient or public partners in numerical aspects, do you think the patient or public partners need to have particular experience or skills?	1: yes<br><br>2: no
If you answered "Yes" to the last questions, what kind of experience or skills are you looking for?	Open question

Theoretical domains framework questions

**Questions**	**Response options**
On a scale of 1 to 7, how familiar are you with involving patient or public partners in numerical aspects of trials ? Scale 1-7, 1 being not at all familiar and 7 being very familiar	1-7
What do you know about how to involve patient or public partners in numerical aspects of trials? Please provide examples of when this has worked well.	Open question
On a scale of 1 to 7, grade your own ability to involve patient or public partners in numerical aspects of trials? Scale 1 – 7: 1 being unable and 7 being very able	1-7
Is involving patient or public partners in numerical aspects of trials an expected role within your job? E.g. an explicit goal or objective within your job specification	1: yes<br><br>2: no<br><br>0: other

For peer review only - http://bmjopen.bmj.com/site/about/guidelines.xhtml

For peer review only

On a scale of 1 to 7, do you feel confident in your ability to involve patient or public partners in numerical aspects of trials? Scale 1- 7, 1 being unable and 7 being very able	1-7
Do you think that involving patient or public partners in numerical aspects of trials is hard to deliver?	1: yes<br><br>2: no<br><br>0: other
If you answered "Yes", what it is that makes it hard and what could make it easier?	Open question
On a scale of 1 to 7, how much do you agree with this sentence? Involving patient or public partners in numerical aspects of trials is a good thing. Scale 1 – 7, 1 being do not agree and 7 being completely agree	
**Do you think involving patient or partners in numerical aspects of a trial can have a positive impact on the following groups?**	
• On the researcher	1: Yes – positive impact<br><br>2: No – no impact<br><br>3: No – negative impact
• On the quality of trial	1: Yes – positive impact<br><br>2: No – no impact

For peer review only - http://bmjopen.bmj.com/site/about/guidelines.xhtml

	3: No – negative impact
• On the patient partner	1: Yes – positive impact<br><br>2: No – no impact<br><br>3: No – negative impact
If you answer "No" to one of the questions, please specify why you think public or patient partners in numerical aspects of trials have no impact or a negative impact	Open question
What positive impacts can patient or public partners have on the numerical aspects of trials?	Open question
Do you think that there is a good balance between the challenges of involving patient or public partners in numerical aspects of trials (e.g. the difficulty of operationalization or implementation) and the potential benefits of patient or public partners involvement?	1: yes<br><br>2: no
**When you involve patient or public partners in numerical aspects of trials, do you get a reward or recognition from the following groups?**	
• Patient and public partners	1: yes<br><br>2: no
• My work peers	1: yes<br><br>2: no

For peer review only - http://bmjopen.bmj.com/site/about/guidelines.xhtml

• My line manager	1: yes<br><br>2: no
Please tell us who else rewards or recognizes you when you involve public and patient partners in numerical aspects of trials	Open question
Will you involve patient or public partners in numerical aspects in your next project?	1: yes definitely<br><br>2: yes probably<br><br>3: no definitely<br><br>4: no probably<br><br>5: I don't know
On a scale of 1 to 7, how much do you agree with this sentence? Working on something else on my agenda is a higher priority or more urgent than involving patient or public partners in the numerical aspects of trials.	1-7
On a scale of 1 to 7, how much do you agree with this sentence? Involving patient or public partners in numerical aspects of trials is something I do automatically.	1-7
Do you have the resources you need to involve patient or public partners in numerical aspects of trials?	1: yes<br><br>2: no
**Do you agree with these sentences?**	
• University/Employer provides training to involve patient or public partners in numerical aspects of trials.	1: yes<br><br>2: no
• Involving patient or public partners in numerical aspects of trials is compatible with daily practice	1: yes

For peer review only

For peer review only - http://bmjopen.bmj.com/site/about/guidelines.xhtml

	2: no
• Patient or public partners are motivated to get involved in numerical aspects of trials	1: yes<br><br>2: no
What in your work environment could help to involve patient or public partners in numerical aspects of trial?	Open question
What in your work environment could be a barrier to involve patient or public partners in numerical aspects of trial?	Open question
On a scale of 1 to 7, how much do you agree with this sentence? Most people who are important (E.g. co-workers, peers) think that I should involve patient or public partners in numerical aspects of trials	1-7
**From the list below, who encourages you to involve patient or public partners in numerical aspects of trials? Tick all the relevant boxes no maximum**	
• Myself	
• Co-workers	
• Manager	
• Scientific literature	
• Social media	
• My personal environment	
• Public	
• Institution	

For peer review only - http://bmjopen.bmj.com/site/about/guidelines.xhtml

• Regulation	
• Mass media	
• No encouragement	
• Other	
**On the list below, who is a barrier for you involve patient or public partners in numerical aspect of trials?**	
• Myself	
• Co-workers	
• Manager	
• Scientific literature	
• Social media	
• My personal environment	
• Public	
• Institution	
• Regulation	
• Mass media	
• No encouragement	

For peer review only - http://bmjopen.bmj.com/site/about/guidelines.xhtml

For peer review only

• Other	
Does involving patient or public partners in numerical aspects of trials lead to any of the following emotions … Tick the relevant boxes (3 boxes minimum)	
Optimism	
Ease	
Calm	
Relaxation	
Appreciation	
Happiness	
Nervousness	
Pessimism	
Depression	
Agitation	
Sadness	
Anxiety	
Do you have a clear plan on which numerical aspects of trials you should involve the patient or partners with?	1: yes<br>2: no

For peer review only

Do you have a clear plan on how you will involve the patient or public partners in the numerical aspects of trials?	1: yes<br><br>2: no
Do you have a clear plan on how often you will involve the patient or public partners in numerical aspects of trials?	1: yes<br><br>2: no
Is there anything that would remind you to involve patients or public partners in numerical aspects of trials?	Open question
On a scale of 1 to 7, how much do you agree with this sentence? I involve patient or public partners in numerical aspects of trials without having to consciously thinking about it. Scale 1-7, 1 being not at all and 7 being definitely	1-7
Is there anything else you would like to tell us about experiences with public involvement in numerical aspects of trial?	Open question

For peer review only

For peer review only - http://bmjopen.bmj.com/site/about/guidelines.xhtml

Reporting checklist for cross sectional study.

Based on the STROBE cross sectional guidelines.

Instructions to authors

Complete this checklist by entering the page numbers from your manuscript where readers will find each of the items listed below.

Your article may not currently address all the items on the checklist. Please modify your text to include the missing information. If you are certain that an item does not apply, please write "n/a" and provide a short explanation.

Upload your completed checklist as an extra file when you submit to a journal.

In your methods section, say that you used the STROBE cross sectionalreporting guidelines, and cite them as:

von Elm E, Altman DG, Egger M, Pocock SJ, Gotzsche PC, Vandenbroucke JP. The Strengthening the Reporting of Observational Studies in Epidemiology (STROBE) Statement: guidelines for reporting observational studies.

		Reporting Item	Page Number
**Title and abstract**			
Title	#1a	Indicate the study’s design with a commonly used term in the title or the abstract	1
Abstract	#1b	Provide in the abstract an informative and balanced summary of what was done and what was found	1
**Introduction**			
Background / rationale	#2	Explain the scientific background and rationale for the investigation being reported	3
Objectives	#3	State specific objectives, including any prespecified hypotheses	3
**Methods**			
Study design	#4	Present key elements of study design early in the paper	3
Setting	#5	Describe the setting, locations, and relevant dates, including periods of	4

For peer review only

		recruitment, exposure, follow-up, and data collection	
Eligibility criteria	#6a	Give the eligibility criteria, and the sources and methods of selection of participants.	4
	#7	Clearly define all outcomes, exposures, predictors, potential confounders, and effect modifiers. Give diagnostic criteria, if applicable	3-4
Data sources / measurement	#8	For each variable of interest give sources of data and details of methods of assessment (measurement). Describe comparability of assessment methods if there is more than one group. Give information separately for for exposed and unexposed groups if applicable.	3-4
Bias	#9	Describe any efforts to address potential sources of bias	NA
Study size	#10	Explain how the study size was arrived at	3-4
Quantitative variables	#11	Explain how quantitative variables were handled in the analyses. If applicable, describe which groupings were chosen, and why	4-5
Statistical methods	#12a	Describe all statistical methods, including those used to control for confounding	4-5
Statistical methods	#12b	Describe any methods used to examine subgroups and interactions	4-5
Statistical methods	#12c	Explain how missing data were addressed	4-5
Statistical methods	#12d	If applicable, describe analytical methods taking account of sampling strategy	4-5
Statistical methods	#12e	Describe any sensitivity analyses	4-5
**Results**			
Participants	#13a	Report numbers of individuals at each stage of study—eg numbers potentially eligible, examined for eligibility, confirmed eligible, included in the study, completing follow-up, and analysed. Give information separately for for exposed and unexposed groups if applicable.	5, Fig 1
Participants	#13b	Give reasons for non-participation at each stage	NA
Participants	#13c	Consider use of a flow diagram	5, Fig 1

For peer review only

Descriptive data	#14a	Give characteristics of study participants (eg demographic, clinical, social) and information on exposures and potential confounders. Give information separately for exposed and unexposed groups if applicable.	p5, Table 1
Descriptive data	#14b	Indicate number of participants with missing data for each variable of interest	p5, Tables 1,2
Outcome data	#15	Report numbers of outcome events or summary measures. Give information separately for exposed and unexposed groups if applicable.	Table 2
Main results	#16a	Give unadjusted estimates and, if applicable, confounder-adjusted estimates and their precision (eg, 95% confidence interval). Make clear which confounders were adjusted for and why they were included	NA
Main results	#16b	Report category boundaries when continuous variables were categorized	NA
Main results	#16c	If relevant, consider translating estimates of relative risk into absolute risk for a meaningful time period	NA
Other analyses	#17	Report other analyses done—e.g., analyses of subgroups and interactions, and sensitivity analyses	NA
**Discussion**			
Key results	#18	Summarise key results with reference to study objectives	9-11
Limitations	#19	Discuss limitations of the study, taking into account sources of potential bias or imprecision. Discuss both direction and magnitude of any potential bias.	11
Interpretation	#20	Give a cautious overall interpretation considering objectives, limitations, multiplicity of analyses, results from similar studies, and other relevant evidence.	9-11
Generalisability	#21	Discuss the generalisability (external validity) of the study results	9-11
**Other Information**			
Funding	#22	Give the source of funding and the role of the funders for the present study and, if applicable, for the original study on which the present article is based	12

Notes:

For peer review only

- 14a: p5, Table 1
- 14b: p5, Tables 1,2 The STROBE checklist is distributed under the terms of the Creative Commons Attribution License CC-BY. This checklist was completed on 14. November 2020 using https://www.goodreports.org/, a tool made by the EQUATOR Network in collaboration with Penelope.ai

For peer review only
